# Family Malvaceae: a potential source of secondary metabolites with chemopreventive and anticancer activities supported with *in silico* pharmacokinetic and pharmacodynamic profiles

**DOI:** 10.3389/fphar.2024.1465055

**Published:** 2024-10-16

**Authors:** Salma Sameh, Ahmed M. Elissawy, Eman Al-Sayed, Rola M. Labib, Hsueh-Wei Chang, Szu-Yin Yu, Fang-Rong Chang, Shyh-Chyun Yang, Abdel Nasser B. Singab

**Affiliations:** ^1^ Department of Pharmacognosy, Faculty of Pharmacy, Ain-Shams University, Cairo, Egypt; ^2^ Center of Drug Discovery Research and Development, Faculty of Pharmacy, Ain-Shams University, Cairo, Egypt; ^3^ Department of Biomedical Science and Environmental Biology, and PhD Program in Life Sciences, College of Life Science, Kaohsiung Medical University, Kaohsiung, Taiwan; ^4^ School of Pharmacy and Graduate Institute of Natural Products, College of Pharmacy, Kaohsiung Medical University, Kaohsiung, Taiwan; ^5^ School of Pharmacy, College of Pharmacy, Kaohsiung Medical University, Kaohsiung, Taiwan; ^6^ Department of Fragrance and Cosmetic Science, College of Pharmacy, Kaohsiung Medical University, Kaohsiung, Taiwan; ^7^ Department of Medical Research, Kaohsiung Medical University Hospital, Kaohsiung, Taiwan

**Keywords:** cancer, herbal nutraceutical, chemopreventive, Malvaceae, pharmacokinetic, pharmacodynamic, tiliroside

## Abstract

**Introduction:**

Cancer is the second most widespread cause of mortality following cardiovascular disorders, and it imposes a heavy global burden. Nowadays, herbal nutraceutical products with a plethora of bioactive metabolites represent a foundation stone for the development of promising chemopreventive and anticancer agents. Certain members of the family Malvaceae have traditionally been employed to relieve tumors. The literature concerning the chemopreventive and anticancer effects of the plant species along with the isolated cytotoxic phytometabolites was reviewed. Based on the findings, comprehensive computational modelling studies were performed to explore the pharmacokinetic and pharmacodynamic profiles of the reported cytotoxic metabolites to present basis for future plant-based anticancer drug discovery.

**Methods:**

All the available information about the anticancer research in family Malvaceae and its cytotoxic phytometabolites were retrieved from official sources. Extensive search was carried out using the keywords Malvaceae, cancer, cytotoxicity, mechanism and signalling pathway. Pharmacokinetic study was performed on the cytotoxic metabolites using SWISS ADME model. Acute oral toxicity expressed as median lethal dose (LD_50_) was predicted using Pro Tox 3.0 web tool. The compounds were docked using AutoDock Vina platform against epidermal growth factor receptor (EGFR kinase enzyme) obtained from the Protein Data Bank. Molecular dynamic simulations and MMGBSA calculations were performed using GROMACS 2024.2 and gmx_MMPBSA tool v1.5.2.

**Results:**

One hundred forty-five articles were eligible in the study. Several tested compounds showed safe pharmacokinetic properties. Also, the molecular docking study showed that the bioactive metabolites possessed agreeable binding affinities to EGFR kinase enzyme. Tiliroside (25), boehmenan (30), boehmenan H (31), and isoquercetin (22) elicited the highest binding affinity toward the enzyme with a score of −10.4, −10.4, −10.2 and −10.1 Kcal/mol compared to the reference drug erlotinib having a binding score equal to −9 Kcal/mol. Additionally, compounds 25 and 31 elicited binding free energies equal to −42.17 and −42.68 Kcal/mol, respectively, comparable to erlotinib.

**Discussion:**

Overall, the current study presents helpful insights into the pharmacokinetic and pharmacodynamic properties of the reported cytotoxic metabolites belonging to family Malvaceae members. The molecular docking and dynamic simulations results intensify the roles of secondary metabolites from medicinal plants in fighting cancer.

## 1 Introduction

Cancer is considered to be a perilous killer worldwide, with estimates of 28.4 million new cancer cases by the year 2040, representing an increase of 47% from the number of diagnosed cancer cases in 2020 (Hricak et al., 2021). Lifestyle and environmental factors represent 90%–95% of the leading causes of cancer, while 5%–10% are related to inherited genes carrying errors (Anand et al., 2008). Several interrelated complex molecular mechanisms govern the development of this serious disease (Heng et al., 2010). The initiation of abnormal cell growth and division expedites the development of cancer and leads to unlimited cell growth and division, resistance to programmed cell death, development of new blood vessels, and metastasis by invading the surrounding healthy tissues (Hornberg et al., 2006). The underlying pathophysiology of this neoplastic disease is related to genetic factors involving gene mutations leading to cancerous transformations and overexpression or formation of novel oncogenes or stopping the action of tumor suppressor genes; also, epigenetic factors can contribute to the development of this disease by changing the methylation level of the genomic DNA without altering its sequence, leading to epimutations that remain through cell division and are transferred to subsequent generations (Heng et al., 2010). Cancer treatment represents a major challenge to humanity due to several reasons, such as the absence of specificity of conventional cancer treatment with failure to provide a long-standing defense against cancer, the emergence of drug resistance, and the lack of early diagnosis due to the silent nature of cancer leading to metastasis to various regions of the body. Furthermore, the currently used synthetic chemotherapeutic agents elicit toxic and serious side effects due to their actions on both cancer and normal cells (Chakraborty and Rahman, 2012).

In this context, a great demand exists for complementary remedies that can prevent and treat cancer. Herbal medicine is considered a potential source of natural and safe chemopreventive and anticancer candidates that can eradicate cancer cells and arrest their growth and metastasis, leading to the protection of people’s lives, reducing undesired pain, and hastening the control of cancer (Rochaniawan, 2021). Chemoprevention is the use of natural drugs to prevent, reduce, reverse, or delay the development of cancer by acting as a blocking agent through different mechanisms including: reducing the activation of pro-carcinogens into carcinogens. Moreover, reducing the amount of reactive oxygen species and promoting repair pathways represent another possible mechanism. Furthermore, they act as suppressing agents against the signalling pathways that activate cell survival and proliferation (Swetha et al., 2022). It is important to note that natural bioactive compounds found in fruits, vegetables and whole grains play a major role in cancer chemoprevention. Moreover, herbal medicines are considered to be beneficial in cancer treatment when employed with conventional remedies (Yin et al., 2013). Around 25% of all newly approved anti-cancer drugs between 1981 and 2019 had been originated from natural sources (Huang et al., 2021).

Traditional strategies employed to eradicate tumor cells are inefficient, while specialized chemotherapy proved to be effective against certain types of cancer. Molecular targeted therapy represents a fundamental approach in cancer treatment. Previous reports showed that kinases represent promising targets (Min and Lee, 2022; Kumar et al., 2017; Raju et al., 2024). Epidermal growth factor receptor belongs to the tyrosine kinase family and is a chief member. EGFR kinase is overexpressed in several types of cancer, such as glioblastoma, brain, lung, and breast cancer (Saadeh et al., 2018). FDA-approved drugs targeting EGFR kinase receptor are available. However, first-generation drugs such as erlotinib exhibited little activity due to tolerance development owing to mutations in the EGFR kinase target. Moreover, second- and third-generation drugs exhibited serious side effects. Therefore, there is a great need to find safe and effective alternatives (Sepay et al., 2022).

The family Malvaceae is commonly known as the Hibiscus, Mallow, or Cotton family. It is a family of flowering plants comprising 244 genera and 4,225 species. Most species are herbs, shrubs, and some are trees. The family members are widely distributed in tropical and temperate regions (Rahman and Gondha, 2014). Some members of family Malvaceae were employed in traditional medicine to relieve inflammation and tumors such as *Corchorus olitorius*, *Abutilon indicum*, *Hibiscus sabdariffa* and *Pavonia xanthogloea* (Oboh et al., 2009; Abat et al., 2017; Mostardeiro et al., 2014). Moreover, it was previously reported that a number of bioactive compounds belonging to several phytochemical classes, such as carotenoids, phenolic acids, flavonoids, coumarins, alkaloids, lignans, cardiac glycosides, sterols, terpenes and polysaccharides isolated from members of the family Malvaceae elicited potential cytotoxic and anticancer activities (Abdel-Razek et al., 2023; Wesley et al., 2013; Hayaza et al., 2019; Moujir et al., 2007; Ahmed et al., 2011; Atolani et al., 2019; Hsu et al., 2015).

However, very few medicinal plants and isolated compounds from the family Malvaceae have been subjected to *in vitro* and *in vivo* cytotoxicity studies. Moreover, data about their possible anticancer mechanisms are scarce. Hence, the objective of this study is to shed light on family Malvaceae members possessing anticancer activity. Also, it is an attempt to comprehensively explore the possible anticancer mechanisms of the isolated bioactive compounds. Additionally, the study provides pharmacokinetic and pharmacodynamic profiles concerning the reported bioactive cytotoxic metabolites to be a starting point for future in depth investigations aiming to develop promising chemopreventive and anticancer candidates.

## 2 Materials and methods

### 2.1 Inclusion criteria

All available information concerning the current status of anticancer research in family Malvaceae members and the cytotoxic activity of the reported metabolites was obtained from official sources such as Pubmed, ScienceDirect, Google Scholar, Reaxys and Springer Link. The keywords employed to retrieve the data were Malvaceae, cancer, cytotoxicity, mechanism and signalling pathway.

### 2.2 Pharmacokinetic profiling, drug likeness and acute oral toxicity study

The pharmacokinetic profiling of the reported bioactive cytotoxic compounds and the reference drug erlotinib was made using SWISS ADME model, which is a free web tool able to predict the ADME profile (http://www.swissadme.ch). Physicochemical properties of the compounds such as topological polar surface area (TPSA), number of hydrogen bond acceptors (HBA), number of hydrogen bond donors (HBD), and molecular weight were evaluated. Moreover, the lipophilicity of the compounds expressed as Log P and their solubility were predicted using SWISS ADME. Important pharmacokinetic properties, including blood-brain barrier permeability (BBB), passive gastrointestinal absorption (HIA) and P-glycoprotein substrate were estimated using BOILED-Egg chart. Additionally, the predicted inhibitory effect of the compounds on CYP 3A4 was assessed. The drug likeness of the compounds was evaluated using the Lipinski’s rule-of-5. Moreover, the SWISS ADME Bioavailability Radar map revealed the drug likeness and the bioavailability of the tested molecules (Daina et al., 2017). Pro Tox 3.0 web server was employed to predict the acute oral toxicity (LD_50_) and the toxicity class (https://tox.charite.de/protox3/) (Banerjee et al., 2024).

### 2.3 *In silico* molecular docking study

Screening of the bioactive compounds was carried out through the AutoDock Vina platform (Trott and Olson, 2010; AbdelRazek et al., 2023). The crystal structure of the target EGFR kinase having the PDB ID (8A27) was obtained from the Protein Data Bank (PDB) (http://www.rcsb.org). Erlotinib was used as the reference drug (Zubair and Bandyopadhyay, 2023). Validation of the docking protocol was carried out by docking the co-crystallized ligand in the receptor, then, calculation of RMSD by comparing the co-crystallized pose and the docked pose. The RMSD value was equal to 0.414 A° which demonstrates the validity of the method. The binding interactions were created through Protein-Ligand Interaction Profiler web tool (http://plip-tool.biotec.tu-dresden.de) and visualized using PyMOL (Adasme et al., 2021).

### 2.4 Molecular dynamic simulations and molecular mechanics generalized-born surface area (MM-GBSA) calculations

Molecular dynamic simulations were performed using GROMACS 2024.2 (Abraham et al., 2015). Protein-ligand complexes were solvated in the TIP3P explicit water model’s cubic box (Berendsen et al., 1984). The system was neutralized by adding 0.1 M concentration of NaCl. The energy of the system was minimized by applying the steepest descent minimization algorithm with a convergence set at 10 kJ/mol and 50,000 steps. Each NVT was run at 300 K temperature and 1 atm pressure for 500 ps, followed by NPT equilibration. At NPT ensembles, 50-ns runs were done. V-rescale modified Berendsen thermostat was used for temperature coupling for each equilibration run. Additionally, pressure coupling was done employing a 2 ps time constant Berendsen coupling (Berendsen et al., 1984; Bussi et al., 2007). Parrinello-Rahman pressure coupling technique was employed during the production runs (Parrinello and Rahman, 1981). Verlet cutoff approach was utilized with 1.2 cutoff and 1.0 nm switch list distances for Van der Waals computations and searching for neighboring atoms. Long-range electrostatics at 1.2 nm was calculated using Particle Mesh Ewald technique (Darden et al., 1993). The bond lengths were restricted by the LINear Constraint Solver (LINCS) algorithm (Hess et al., 1997). The topology and parameters of the protein molecules were constructed using CHARMM36 all-atom force field. Ligand parameters were obtained from SwissParam server. The RMSD and RMSF of the lead phytochemical-bound, standard inhibitor-bound were estimated using the gmx_rms and gmx_rmsf options of gromacs, respectively. The Rg was determined using the gmx_gyrate. The energy calculations were driven employing the gmx_energy tool. Hydrogen bond formation was assessed using the gmx_hbond tool. Grace Software was employed to make graphical representations (https://plasma-gate.weizmann.ac.il/Grace/). The binding free energy of the top hits and reference compounds was calculated by the MMGBSA method using the gmx_MMPBSA tool v1.5.2 (Valdés-Tresanco et al., 2021). The tool was conjugated with GROMACS to make computations based on AMBER (Miller et al., 2012).

## 3 Results

### 3.1 Members of family Malvaceae possessing anticancer activity

The anticancer activity of 24 species from family Malvaceae is stated in this study. It is important to note that plant extracts with IC_50_ less than 20 μg/mL are considered to possess potent *in vitro* cytotoxic activity according to the criteria of the US National Cancer Institute (NCI). Moreover, crude extracts with IC_50_ up to 30 μg/mL are suggested to be promising for further purification according to NCI (Canga et al., 2022). The extracts and fractions prepared from the different plants and possessing potent activity are summarized in Supplementary Table S1, while the plants’ extracts and fractions exhibiting moderate or weak effect are listed in Supplementary Table S2.

#### 3.1.1 *Abelmoschus esculentus* L.

By reviewing the literature, we observed that the different organs of *Abelmoschus esculentus* exerted promising cytotoxic activity against diverse types of cancer. It was reported that gold nanoparticles (AU NPS) prepared from the aqueous pulp extract of red okra were tested *in vitro* against Jurkat (human acute myeloid leukemia), K562 (human chronic myeloid leukemia) and DL (Dalton’s lymphoma) cell lines. Results showed that the tested gold nanoparticles at concentrations of 5, 10, 25 and 50 μg/mL elicited remarkable inhibition in viability of Jurkat cells by 45.1, 48.6, 81.3, and 87.2%, respectively, with IC_50_ equal to 8.17 μg/mL. The gold nanoparticles possessed potent activity against Jurkat cells, which was attributed to an increase in reactive oxygen species and alteration of the mitochondrial membrane potential leading to apoptosis. However, this study proved that Au NPS exerted moderate antiproliferative activity against the K562 and DL cell lines (Mollick et al., 2014; Petruzzello, 2022).

Another study proved that silver nanoparticles (Ag NPS) synthesized from aqueous extract prepared from flowers exhibited potential *in vitro* cytotoxic activity against lung cancer cell lines (A549) and mesenchymal cell lines (TERT-4) with IC_50_ = 1.74 and 1.65 μg/mL, respectively, compared to 5-fluorouracil having IC_50_ = 0.763 and 0.781 μg/mL, respectively*.* The observed cytotoxic activity is due to the ability of silver nanoparticles to enter the cells *via* endocytosis and pinocytosis, promoting the elevation of reactive oxygen species inside the cells, leading to DNA deterioration and apoptosis. Furthermore, the bioactive constituents coating the silver nanoparticles elevate oxidative stress and activate caspase-mediated and mitochondria-dependent pathways leading to cell death. Also, it was observed that there were changes in the expression of anti-apoptotic (Bcl-2) and pro-apoptotic (Bax) genes and stimulation of caspase-3 and caspase-9 (Devanesan and AlSalhi, 2021). Moreover, the ethyl acetate fraction obtained from the flowers exerted weak cytotoxic activity against HepG2 cells (Solomon et al., 2016).

Furthermore, the *in vivo* anticancer activity of the ethanol extract prepared from the pods of red okra was tested against *N*-methyl-*N*-nitrosourea (MNU) induced breast cancer model in rats. The findings showed that the extract at doses of 100 and 200 mg/kg b. w. produced potent anticancer effects by regulating the immune response by diminishing the interleukin (IL)-6, IL-1*ß*, tumor necrosis factor (TNF)-*α*, IL-17, IL-10, and tumor growth factor-*β*. Additionally, it was observed that the extract at a dose of 200 mg/kg b. w. promoted the activation of CD4^+^ and CD8^+^ T cells and arrested the generation of epithelial cells in the mammary glands (Abdel-Razek et al., 2023; Pramudya et al., 2022).

Moreover, a polysaccharide extract of fruits prepared by ethanol extraction showed weak activity against human liver cancer cell lines (Huh7it) by inducing 5.62% and 5.82% early and late apoptosis, respectively at concentration of 600 μg/mL. Additionally, cell cycle arrest at G_0_/G_1_ phase was observed (Hayaza et al., 2019). A further study showed that cerium oxide nanoparticles (CeO_2_) prepared from the fruits exerted weak *in vitro* cytotoxic activity against HeLa cells (Ahmed et al., 2021).

An *in vitro* study was conducted to test the antiproliferative effect of lectin isolated from the seeds against U87 glioblastoma cell lines. Results showed that the compound exerted pronounced cytotoxic activity against the tested cell line with IC_50_ equal to 21 μg/mL*.* The findings revealed that cells treated with lectin at a concentration of 21 μg/mL demonstrated an increase in late apoptotic cells (Annexin and PI + ve) by 68%, which was comparable to temozolomide standard-treated cell lines, which exhibited a 75% increase in late apoptotic cells. Moreover, lectin-treated cell lines showed 16% Annexin V FITC-positive U87 cells. Thus, early and late apoptosis were induced. Also, nuclear morphological alterations and apoptosis were observed when utilizing acridine orange and ethidium bromide on treated cell lines with 21 μg/mL. The findings revealed that cells were stained green and red, respectively, indicating DNA damage and cell death. Moreover, it was deduced that apoptosis was induced by the ability of lectin to promote the generation of caspase-3 and caspase-7. Furthermore, lectin induced cell cycle arrest at G_0_/G_1_ phase, leading to apoptosis (Musthafa et al., 2021).

#### 3.1.2 *Abelmoschus moschatus* Medik.

Reports have shown that the leaves, seeds, and aerial parts of *Abelmoschus moschatus* possess cytotoxic activity. The ethanol and acetone extracts prepared from the aerial parts exhibited moderate *in vitro* cytotoxic activity against HepG2 cells (Sebastian et al., 2022). Moreover, hydroalcohol extracts prepared from the leaves and seeds of the plant elicited weak *in vitro* cytotoxic activity against colorectal adenocarcinoma (COLO-205) and retinoblastoma (Y79) cell lines (Gul et al., 2011; Rojas-Sandoval, 2018).

#### 3.1.3 *Abutilon grandiflorum* G. Don

The petroleum ether, ethyl acetate, and aqueous extracts prepared from the roots of *Abutilon grandiflorum* were tested *in vitro*. The results showed that the ethyl acetate extract exerted moderate cytotoxic activity against colon cell line HT-29, while the petroleum ether and aqueous extracts showed low activity (Beha et al., 2004).

#### 3.1.4 *Abutilon hirtum* Lam.

An *in vitro* study revealed that the petroleum ether extract prepared from the leaves of *Abutilon hirtum* when tested at concentrations of 100 and 50 μg/mL exhibited 100% reduction in Ehrlich ascites carcinoma tumor cells (Ha, 2001). Another *in vitro* study showed that the aqueous extract prepared from the flowers elicited weak cytotoxic activity against MCF-7 cells (Wesley et al., 2013).

#### 3.1.5 *Abutilon indicum* L.

The petroleum ether and methanol extracts along with two ethyl acetate subfractions (AIM-C and AIM-E) obtained from the methanol extract prepared from the leaves exhibited moderate activity against U87MG glioblastoma cell lines (Khan et al., 2015). Moreover, the methanol extract prepared from the whole plant of *Abutilon indicum* exerted very weak cytotoxic activity against human melanoma cell lines (SK-MEL28) and human non-small cell lung cancer cell lines (NCI-H23) (Srikanth et al., 2012).

#### 3.1.6 *Adansonia digitata* L.

Different organs of *Adansonia digitata* elicited promising cytotoxic activity against various types of cancer. The brine shrimp lethality bioassay was conducted using the methanol extract, petroleum ether soluble fraction, chloroform soluble fraction, and aqueous soluble fraction prepared from the leaves and barks of *Adansonia digitata*. The results showed that the leaves petroleum ether soluble fraction and the bark methanol extract exhibited the highest activity expressing IC_50_ equal to 0.284 and 6.99 μg/mL, respectively, compared to vincristine sulphate having IC_50_ = 0.44 μg/mL (Tahia et al., 2015). In addition, the seeds aqueous ethanol extract was evaluated *in vitro* against lung cancer cells (A549), breast cancer cells (MCF7), hepatic cancer cells (HepG2), normal cell line melanocytes cells (HFB4) and fibrinocytes cells (BHK). The results showed that the extract exhibited an IC_50_ = 11.5 μg/mL against MCF-7 cells. Moreover, it elicited a remarkable activity against HFB4 and BHK normal cells with IC_50_ = 11.6 and 24.5 μg/mL, respectively (Abd Alaziz and Abdelmageed, 2019).

Moreover, the silver nanoparticles (Ag NPS) prepared from the fruits and the fruit aqueous extract exerted moderate *in vitro* cytotoxic activity against HTC116 and SW480 colon cancer cell lines. It was observed that the fruits Ag NPS exhibited higher activity against both cell lines compared to the fruits extract. It was supposed that the cytotoxic activity was due to the reduction in the expression of CTNNB1 and LRP6 genes, which in turn reduces cell proliferation and delays tumor expansion. Also, it was observed that the level of LRP5 genes was very high in both treated cell lines (Almukaynizi et al., 2022).

#### 3.1.7 *Althaea officinalis* L.

An *in vitro* study proved that the crude, flavonoid and phytosterol extracts of *Althaea officinalis* exhibited moderate cytotoxic activity against breast cancer cell lines (AMJ 13) (Kadhum et al., 2021). Furthermore, it was previously reported that the aqueous extract prepared from the flowers elicited weak *in vitro* cytotoxic activity against A549, EB, HCT116 p53 null cells, p53 wild type HCT116, MCF-7 and HeLa 229 cell lines (Farhat et al., 2022).

#### 3.1.8 *Althaea rosea* (L.) Cav.

It was observed that *Althaea rosea* could be a good nucleus for developing chemopreventive and anticancer drugs as it exerts its action by inhibiting the cancerous cell transformation through reducing the activity of kinase in EGFR receptor. Also, it reduced cell generation produced by EGF in murine embryonic fibroblasts having EGFR (Choi et al., 2012).

#### 3.1.9 *Brachychiton discolor* F. Muell.

The cytotoxic activity of the essential oils obtained from the leaves and flowers of *Brachychiton discolor* was tested against MCF-7, A549, and HepG2 cell lines. It was observed that the essential oils from the flowers exhibited potent cytotoxic activity with IC_50_ = 10.9 and 7.98 μg/mL against MCF-7 and A549 cells, respectively, compared to doxorubicin having IC_50_ = 0.43 and 0.91 μg/mL, respectively (Thabet et al., 2020).

#### 3.1.10 *Brachychiton populneus* (Schott and Endl.) R. Br.

The silver nanoparticles (Ag NPS) prepared from the leaves aqueous extract of *Brachychiton populneus* exhibited weak cytotoxic activity against Uppsala 87 malignant glioma (U87 MG) and human embryonic kidney cell lines (HEK 293) (Naveed et al., 2022).

#### 3.1.11 *Brachychiton rupestris* (T. Mitch. ex Lindl.) K. Schum.

An *in vitro* study showed that the leaves essential oils exerted pronounced activity against HepG2 cell lines with IC_50_ equal to 8.57 μg/mL compared to doxorubicin (IC_50_ = 0.51 μg/mL) (Thabet et al., 2020).

#### 3.1.12 *Cola gigantea* A. Chev.

The *in vitro* cytotoxic activity of the essential oils obtained from the seeds of *Cola gigantea* was evaluated against human foreskin fibroblast (HFF) at doses ranging from 0 to 1,000 μg/mL. The oil exhibited potent activity with IC_50_ less than 10 μg/mL (Atolani et al., 2019).

#### 3.1.13 *Corchorus olitorius* L.

Several organs of *Corchorus olitorius* exhibited promising cytotoxic activity. It was reported that the aqueous ethanol extract prepared from the aerial parts showed pronounced effects due to the induction of apoptosis with IC_50_ (8.6, 10.3 and 5.56 μg/mL) against metastatic melanoma (CaCi 1962 and LiGh 1927B) and human melanoma SK-MEL28 cell lines, respectively, which were comparable to the standard drug cisplatin (Handoussa et al., 2013). In addition, gold and iron oxide nanoparticles prepared from the leaves 70% ethanol extract exhibited promising effects against MCF-7 cell lines with IC_50_ (6.97 and 5.82 μg/mL), respectively, which were comparable to the standard drug having IC_50_ equal to 3.95 μg/mL (El-Rafie et al., 2016).

In the same context, the hexane, ethyl acetate, methanol, and aqueous extracts prepared from the whole plant were tested against non-cancerous cell lines (L929), breast cancer (MCF-7), and lung cancer cells (A549). It was observed that the methanol extract exerted the most promising activity against MCF-7 and A549 cell lines with IC_50_ equal to 20 and 12.45 μg/mL, respectively, which were comparable to cisplatin having an IC_50_ equal to 2.50 and 13.13 μg/mL, respectively. Also, the extract showed low level of toxicity against non-cancerous L929 cell lines (IC_50_ = 227.84 μg/mL) compared with cisplatin (IC_50_ = 10.34 μg/mL). The results revealed that early and late apoptosis were induced by 4.18% and 5.72%, respectively, in MCF-7 treated cells with methanol extract, while it induced early and late apoptosis by 4.55% and 11.5%, respectively, in A549 cells. Moreover, apoptosis was induced in the treated cell lines with methanol extract by activation of caspase-3. A decrease in Bcl-2 protein level and DNA damage were also observed (Alshabi et al., 2022).

**FIGURE 1:**
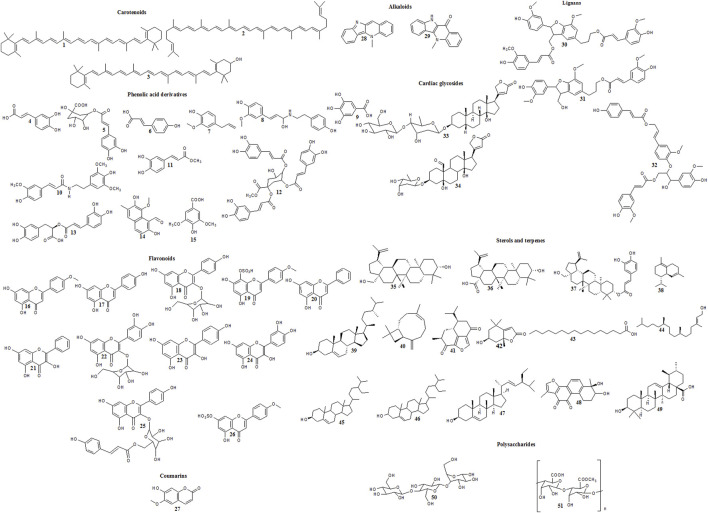
Reported cytotoxic carotenoids, phenolic acid derivatives, flavonoids, coumarins, alkaloids, lignans, cardiac glycosides, sterols, terpenes and polysaccharides in family Malvaceae.

Furthermore, the *in vitro* and *in ovo* cytotoxic and antiangiogenic activities of the aqueous extract prepared from the leaves and the active phytometabolites chlorogenic acid (5) and isoquercetin (22) (Figure 1) were evaluated. They exhibited weak cytotoxic activity against human melanoma (A-375), gastric cancer (AGS), and pancreatic cancer cell lines (SUIT-2). Moreover, all tested substances arrested angiogenesis in the A-375 and AGS cell lines (Tosoc et al., 2021).

Another study showed that methyl-1,4,5-tri-*O*-caffeoyl quinate (12) (Figure 1) isolated from whole plant methanol extract was tested at concentrations reaching 1.6 mM against HeLa cells. It exerted mild cytotoxic effect at 800 µM or higher. Also, an *in silico* molecular docking study showed that the compound possessed pronounced binding affinity toward metalloproteinase-9, fibroblast growth factor receptor 2, and epidermal growth factor receptor involved in tumourigenesis. Despite the weak cytotoxic activity of the compound, analogues with higher potency could be developed after carrying out structure-activity relationship studies (Taiwo et al., 2016).

#### 3.1.14 *Hibiscus cannabinus* L.

The *in vitro* cytotoxic activities of lignans isolated from the acetone extract prepared from the bark of *Hibiscus cannabinus* were tested against human carcinoma of the cervix (HeLa), human carcinoma of the larynx (Hep-2), and human lung carcinoma cells (A549). Two lignans, boehmenan H (31) and *threo*-carolignan K (32) (Figure 1), elicited strong effects against all tested cell lines (Moujir et al., 2007).

#### 3.1.15 *Hibiscus ficulneus* L.

Reports showed that the lignan compound, namely boehmenan (30) isolated from the stem methanol extract exhibited a good cytotoxic activity against Wnt-independent (RKO) and Wnt-dependent cells (HCT116), respectively (Shono et al., 2015).

#### 3.1.16 *Hibiscus sabdariffa* L.

Ethanol extract prepared from the calices of *Hibiscus sabdariffa* exerted weak cytotoxic activity against MCF-7 and MDA-MB231 cell lines. The observed activity was attributed to the ability of the fraction to stimulate the reduction in proteasome activity and promote the autophagic activity in both cell lines. Moreover, in MCF-7, the levels of mRNA and protein of ER*α* were remarkably decreased in treated cells, leading to dysregulation in ER*α* and a decrease in the protein levels of its two targets, namely BRCA1 and CAV1. The reduction in BRCA1 led to a decrease in the repair of DNA damage leading to apoptosis and cell death. Also, the decrease in CAV1 led to a reduction in cell migration and invasion (Malacrida et al., 2022).

#### 3.1.17 *Hibiscus syriacus* L.

Previous reports showed that *Hibiscus syriacus* is rich with carotenoids and triterpenes possessing promising cytotoxic activity (Hsu et al., 2015; Azimova et al., 2011). By reviewing the literature, it was observed that the triterpenoid betulin-3-caffeate (37) (Figure 1) elicited potent *in vitro* cytotoxic activity against lung cancer cell lines (A549) (Shi et al., 2014).

Furthermore, the *in vitro* and *in vivo* anticancer activity of a callus extract prepared from the leaves of *Hibiscus syriacus* was tested. The *in vitro* results showed that the extract possessed moderate activity against HT-29 cell lines. Moreover, the *in vivo* study carried out in thymus-deficient mice bearing HT-29 xenografts revealed that the extract elicited good anticancer activity at doses of 100 and 200 mg/kg when used for 15 days through notch signalling-mediated suppression of cholesterol synthesis. Thus, it was deduced that the extract possessed good anti-colorectal cancer activity (Xu et al., 2022).

#### 3.1.18 *Hibiscus tiliaceus* L.

Reports showed that the isolated compounds hibiscusamide (10) and *N*-*trans*-feruloyltyramine (8) (Figure 1) from the stem wood methanol extract possessed strong *in vitro* cytotoxic activity against leukemia cell lines (P-388) and human colon cancer cell lines (HT-29) (Chen et al., 2006). Moreover, the ethyl acetate extract prepared from the leaves exhibited potent cytotoxic activity against breast cancer cell lines (MCF-7) with IC_50_ equal to 10 μg/mL by inducing early and late apoptosis (Andriani et al., 2020).

#### 3.1.19 *Malva parviflora* L.

It was previously reported that the stems of *Malva parviflora* are rich with phenolic and flavonoid compounds (Singh, 2017). Reports showed that the hexane extract prepared from the stems exerted weak *in vitro* cytotoxic activity against MDA-MB231 cell lines (Nasr et al., 2018).

#### 3.1.20 *Malva sylvestris* L.

The iron oxide nanoparticles prepared from *Malva sylvestris* possessed weak *in vitro* cytotoxic activity against MCF-7 and HepG2 cell lines (Mousavi et al., 2020).

#### 3.1.21 *Pavonia odorata* willd.

An *in vitro* study proved that the methanol extract prepared from the whole plant of *Pavonia odorata* exerted weak cytotoxic activity against human breast (MD-MB231), prostate cancer (PC3), and lung cancer cell lines (Calu-6). It was deduced that the present flavonoids were responsible for the antiproliferative activity by reducing the Na^+^ and K^+^ ATPase in the plasma membrane, leading to the inhibition of the elevated glycolytic activity of the tumor cells (Girish et al., 2016).

#### 3.1.22 *Pavonia xanthogloea* Ekman

The aqueous fraction prepared from the aerial parts of *Pavonia xanthogloea* elicited weak cytotoxic activity against human lymphocyte cells (Mostardeiro et al., 2014).

#### 3.1.23 *Sida acuta* Burm. F.

Reports showed that cryptolepinone (29) (Figure 1) and *N*-*trans*-feruloyltyramine (8) (Figure 1) isolated from the ethyl acetate extract elicited 83.3% and 75% reduction in dimethylbenz [a]-anthracene (DMBA)-induced preneoplastic lesions in mouse mammary glands in organ culture at a dose of 10 μg/mL, respectively. Therefore, it was deduced that those compounds could be used as chemopreventive agents (Jang et al., 2003). Moreover, the alkaloid cryptolepine (28) (Figure 1) isolated from the methanol extract prepared from the whole plant and tested at concentrations of 1.25, 2.5 and 5 µM was able to solve tumor necrosis factor (TNF)-related apoptosis-inducing ligand resistance in human gastric adenocarcinoma (AGS) cell lines. It was observed that cryptolepine stimulated the release of mitochondrial cytochrome-c leading to cell cycle arrest and apoptosis (Ahmed et al., 2011).

#### 3.1.24 *Wissadula periplocifolia* (L.) C. Presl

The *in vitro* cytotoxic activities of the isolated compounds from the ethanol extract aerial parts were evaluated. It was observed that acacetin (16) and tiliroside (25) (Figure 1) exhibited good cytotoxic activity against PC-3M cell lines. Moreover, acacetin (16) elicited remarkable effect against UVW glioma. Furthermore, the combination of 7-*O*-sulphate acacetin (wissadulin) (26) and 4′-*O*-methyl-8-*O*-sulphate isoscutellarein (caicoine) (19) exerted activity against PC-3M with IC_50_ (92.14 μg/mL) (Teles et al., 2015).

### 3.2 Bioactive phytometabolites in family Malvaceae and their chemopreventive and anticancer effects

A number of bioactive secondary metabolites belonging to several chemical classes were identified and isolated from different members of family Malvaceae (Gomaa et al., 2018; Oboh et al., 2012; Abd El-Salam et al., 2016; Hasan and Kadhim, 2018; Yakoub et al., 2018; Ramadevi, 2013; Hassan et al., 2019; Ragasa et al., 2016; Biswas et al., 2022; Arfa, 2022; Deters et al., 2010; Shah et al., 2011). Reports showed that some of them elicited chemopreventive and anticancer activities. The mentioned compounds are listed in Supplementary Table S3 and illustrated in Figure 1.

#### 3.2.1 Carotenoids

Carotenoids are characterized by their polyene structure containing 8–13 conjugated double bonds which are effective in scavenging reactive oxygen and nitrogen species (Saini and Keum, 2019). It exists an inverse relationship between carotenoid intake and the incidence of various types of cancer such as breast, prostate, lung and colorectal cancers (Saini et al., 2020).

*β*-carotene (1), lycopene (2) and zeaxanthin (3) occurred in family Malvaceae (Abdel-Razek et al., 2023; Azimova et al., 2011). Carotenoids exert their anticancer properties through modulating various signalling pathways. It was previously reported that *β*-carotene (1) elicited promising antiproliferative *in vitro* activity against human breast cancer (MCF-7 cells) by activating caspase-3 enzyme, decreasing the expression of the anti-apoptotic protein, Bcl2 and PARP, decreasing the level of NF-κB, inhibiting the activation of ERK1/2, and inhibiting Akt activity, thus leading to apoptosis (Sowmya et al., 2017).

Moreover, lycopene (2) exhibited promising *in vitro* anticancer activity against human colon cancer cells (HT-29) by inhibiting the phosphorylation of Akt, glycogen synthase kinase-3*β* (GSK-3*β*), and ERK 1/2 proteins and by suppressing MMP-7 expression, thus preventing metastasis and angiogenesis (Lin et al., 2011). Moreover, it was reported that lycopene reduced H pylori–associated gastric cancer by decreasing the levels of ROS, inhibiting Jak1/Stat3 activation, eliciting alteration in Wnt/*β*-catenin multiprotein complex molecules, reducing the expression of *c-myc* and cyclin E, and decreasing the cell proliferation in H pylori–infected gastric epithelial cells (Park et al., 2019). Furthermore, lycopene exerted promising *in vitro* activity against prostatic carcinoma cells (LNCaP) by eliciting Ras inactivation, reducing the activation of NF-κB, decreasing the level of ROS, inhibiting the phosphorylation of c-jun N-terminal kinase, extracellular signal-regulated kinase 1/2, and p38. It elicited also a decrease in cyclin D1 and increased p21, p27, p53 levels, and Bax:Bcl-2 ratio, thus causing cell cycle arrest and inducing apoptosis (Palozza et al., 2010).

It was previously reported that zeaxanthin (3) exerted pronounced selective cytotoxic activity against 12 different types of human gastric carcinoma. The compound exerted its action by affecting ROS-mediated MAPK, AKT, NF-κB, and STAT3 signalling pathways. It was observed that it decreased the potential of mitochondrial membrane, elevated the level of cytochrome-c and Bax. Also, it inhibited caspase-3 and PARP expression and reduced the expression of Bcl-2, pro-caspase-3, and pro-PARP, leading to apoptosis. Moreover, it elicited cell cycle arrest at the G2/M phase by elevating p21 and p27 levels and inhibiting AKT, cyclin A, cyclin B1, and cyclin-dependent kinase 1/2 levels. It exerted potent cytotoxic activity against AGS cells with IC_50_ = 17 µM compared to the standard drug 5-fluorouracil having IC_50_ = 23.34 µM (Sheng et al., 2020).

#### 3.2.2 Phenolic acid derivatives

Several phenolic acids possessing promising cytotoxic activity were identified in various species of family Malvaceae. Previous reports proved that phenolic compounds possessed significant anti-carcinogenic activity (Singab et al., 2011). Caffeic acid (4) is available in many foods in the form of ester leading to difficult absorption inside the human body. A small quantity is initially absorbed in the stomach. Following this, cleavage of the ester moiety occurs in the colon to release the free acid. Monocarboxylic acid transporters mediate the active transport of the compound into the intestinal wall. The compound is present at high concentration in the plasma for 1 h after ingestion of foods containing the compound. Then, the plasma concentration decreases rapidly. Therefore, the dose of caffeic acid must be given every 2 h. The compound is detoxified and eliminated through enzymatic conjugation which makes the compound more hydrophilic (Espíndola et al., 2019).

Reports showed that the compound possessed promising *in vitro* and *in vivo* antiproliferative activity through various mechanisms. It prevents the formation of ROS and reduces the oxidative stress through preventing free radical generation by stopping the chain reaction with another different molecule. The free radicals are converted into thermodynamically constant substances by the action of caffeic acid that donates hydrogen/electrons to them. Another way to prevent the formation of free radicals is by stopping peroxides decomposition by acting as chelating agent through the formation of complexes with metals. In addition, it causes apoptotic alterations in cancer cells by acting as pro-oxidant through the induction of lipid peroxidation markers, leading to an increase in ROS and modifications in MMP leading to the damage of DNA in cancer cells. Furthermore, it induces cell cycle arrest and promotes cell death owing to its apoptotic properties as it inhibits the Bcl-2 activity, leading to cytochrome-c release and enhancement of caspase-3 activity. Also, it inhibits angiogenesis and metastasis through decreasing HIF-1*α* activity, leading to a reduction in JNK-1 phosphorylation which in turn leads to stopping of VEGF-mediated proliferation and decreases cancer growth. Also, it decreases the expression of MMP-2 and MMP-9 (Alam et al., 2022). An *in vitro* study was conducted to evaluate the antiproliferative activity of caffeic acid using MTT assay against breast carcinoma cells (MCF-7). The MCF-7 cells were treated with different concentrations of the compound ranging from 5 to 200 μg/mL for 48 and 72 h. Tamoxifen citrate was employed as the standard drug. The results revealed that the compound exhibited IC_50_ = 170 and 159 μg/mL in treated cells for 48 and 72 h, respectively, compared to the standard drug having IC_50_ = 19.4 and 16 μg/mL, respectively. The observed activity of caffeic acid was related to alterations in the expression of p53, p21 and Mcl-1 genes responsible for apoptosis. Moreover, the increased expression of p21 inhibited CDK2, CDK3, and CDK4, leading to cell cycle arrest in G1 or G2 phases (Rezaei-Seresht et al., 2019).

Furthermore, it exhibited potent cytotoxic activity against liver cancer cell lines (Huh-7) with IC_50_ = 3 μg/mL (El-Din et al., 2014). Another study showed that the combination of caffeic acid (50 µM) and cisplatin (5 µM) led to 1.7 fold enhancement in caspase action, leading to an increase in the apoptotic cascade in human ovarian carcinoma cells (A2780 cis cells) (Alam et al., 2022). Additionally, an *in vivo* study was conducted to test the effect of the administration of caffeic acid (30 mg) on the efficacy of transarterial embolization (TAE) therapy in rats inoculated with the N1-S1 hepatocellular carcinoma cell line. TAE is employed to block blood supply in the arteries, leading to ischemia and depletion of oxygen and nutrients in tumor cells. The results showed the efficacy of caffeic acid as it reduced tumor volume by 70%–85% compared to the treated group with TAE technique alone. The observed activity was due to the ability of caffeic acid to activate the intrinsic pathway of apoptosis and cause DNA fragmentation leading to destroying cancer cells (Wilkins et al., 2017). Moreover, the compound has been subjected to clinical trials with codes NCT03070262 and NCT04648917 entitled (The Efficacy and Safety of Caffeic Acid for Esophageal Cancer) and (GASC1 Inhibitor Caffeic Acid for Squamous Esophageal Cell Cancer [ESCC]), respectively. However, no results have yet been published (Cortez et al., 2024).

Moreover, chlorogenic acid (5) exhibited potent *in vitro* and *in vivo* antiproliferative activity against hepatocellular carcinoma by inducing cell cycle arrest in the S phase, reducing the phosphorylation of ERK1/2, and decreasing MMP-2 and MMP-9 expression (Yan et al., 2017). Also, it exerted promising anticancer activity against A549 cell lines by inhibiting the expression of Bcl-2 and by activating Bax and caspase-3 enzyme (Yamagata et al., 2018).

Furthermore, It was reported that *p*-coumaric acid (6) exerted promising *in vitro* antiproliferative activity against colon cancer cells like HT 29 and HCT 15 and caused cell cycle arrest at sub-G1 phase, and induced apoptosis (Jaganathan et al., 2013). Also, it exerted promising *in vivo* anticancer effects by eliciting antiangiogenic effect in endothelial cells by down-regulating the vascular endothelial growth factor, AKT, and ERK signalling pathways (Kong et al., 2013).

Previous studies showed that eugenol (7) elicited cytotoxic activity against PC-3 cell lines and possessed the ability to scavenge the nitric oxide radicals; thus, it could be a lead for developing chemopreventive agents (Sharma et al., 2017).

Reports revealed that *N*-*trans*-feruloyltyramine (8) possessed selective cytotoxic activity and is a good candidate for developing chemopreventive and anticancer medications as it exhibited antiproliferative activity by inhibiting the oxidative damage caused by H_2_O_2_. Also, it conserves the integrity of mitochondrial membrane by reducing morphological alteration in mitochondria (Gao et al., 2019).

Gallic acid (9) is a phenolic compound available in a variety of foods. Bioavailability studies showed that the compound possesses good absorption inside the human body. 4-*O*-methyl gallic acid was the major metabolite appearing in human plasma. After ingestion of 50 mg free gallic acid, a high human plasma concentration of the free acid and 4-*O*-methyl gallic acid is observed (Kaliora et al., 2013). It was assumed that the compound can be administered as oral tablets during cancer therapy. Concerning the toxicity of gallic acid, no adverse effects were observed when administering the compound to mice at a dose of 5,000 mg/kg b. w. Reports showed that gallic acid (9) possessed a strong cytotoxic activity (Sameh et al., 2018). It showed *in vitro* anticancer activity against human hepatoma cells (SMMC-7721) with IC_50_ = 50 μM/L by reducing the cell proliferation and inducing apoptosis by increasing the tumor suppressor gene p53 (Subramanian et al., 2015). Furthermore, it revealed anticancer activity against A375. S2 human melanoma cells by inducing apoptosis through increasing the expression of apoptotic protein Bax and reducing the expression of anti-apoptotic protein Bcl-2 and also through the activation of caspase-9 and caspase-3 (Lo et al., 2010). Moreover, it elicited anticancer effect against cervical cancer cells (HeLa) by increasing the production of reactive oxygen species and reducing the GSH level in HeLa cells (You et al., 2010). Another study showed that it possessed potent activity against MCF-7 cells with IC_50_ = 18 μg/mL compared to the standard drug tamoxifen citrate eliciting an IC_50_ = 16 μg/mL. It was observed that the compound altered the expression of p53, p21, and Mcl-1 genes responsible for apoptosis (Rezaei-Seresht et al., 2019).

Reports showed that hibiscusamide (10) exhibited cytotoxic activity against Hep3B cells by reducing p-STAT3, p-JAK2, and p-ERK phosphorylation and inhibiting IL-6 signalling pathway (Hwang et al., 2016). Furthermore, it was deduced that methyl caffeate (11) could be developed into a supplement used for chemoprevention as it elicits apoptosis by selectively inhibiting 3-phosphoglycerate dehydrogenase (PHGDH) enzyme which is highly expressed in cancer cells (Wang et al., 2021). Moreover, it was reported that methyl-1, 4, 5-tri-*O*-caffeoyl quinate (12) elicited mild cytotoxic activity against HeLa cells (Taiwo et al., 2016).

Rosmarinic acid (13) showed cytotoxic activity against rat glioblastoma C6 cells due to its antioxidant effect and its ability to reduce cell proliferation at concentrations ranging from 80 to 130 μM, while at higher concentration over 200 μM, it stimulates prooxidant activity, leading to necrosis and cell death (Ramanauskiene et al., 2016). Another study showed that the compound elicited promising anticancer effect by inhibiting the microtubule affinity regulating kinase (MARK4) (Anwar et al., 2020). Furthermore, it was reported that it induced apoptosis in HepG2 cells, inhibited proteasome, and induced cellular stress (Ozgun and Ozgun, 2020). Moreover, syriacusin A (14) possessed the ability to reduce cell proliferation and to enhance apoptosis (Μatsumoto et al., 2020).

By reviewing the literature, it was observed that syringic acid (15) represents a promising molecule for developing anticancer drugs as it induced cytotoxic activity in HepG2 cells by enhancing the expression of caspase-3 and -9, cytochrome-c, Apaf-1, Bax, and p53 gene. Also, it reduced the expression of Bcl-2, leading to apoptosis (Gheena and Ezhilarasan, 2019).

#### 3.2.3 Flavonoids

Flavonoids are divided into 6 classes which are isoflavonoids, flavanones, flavanols, flavonols, flavones and anthocyanidins (Kopustinskiene et al., 2020). Several *in vitro* and *in vivo* studies showed that flavonoids possessed promising anticancer activities (Rodríguez-García et al., 2019; Abotaleb et al., 2018; Chirumbolo et al., 2018; Mostafa et al., 2017). Generally, flavonoids act by modulating the redox homeostasis. They act as antioxidant in normal cells, directly by scavenging the reactive oxygen species or indirectly by activating the antioxidant enzymes or arresting the prooxidant enzymes. Moreover, they elicit prooxidant activity in cancer cells, leading to apoptosis (Kopustinskiene et al., 2020). Also, reports showed that flavonoids possessed anti-inflammatory activity (Ayoub et al., 2021; Labib et al., 2013).

Previous studies showed that acacetin (16) exerted antiproliferative activity *in vitro* and *in vivo* animal models. It showed cytotoxic activity against A549, NCI–H187, H1299 cells, leukemia CLL, Jurkat T cells, MOLT-4, HL-60, U937, K562 cells, breast carcinoma cells (MDA-MB-231, MCF-7, MCF-10A), hepatocellular carcinoma (SMMC-7721, HepG2), skin cells (Hs27, HSC-3, B-16), esophageal squamous cell carcinoma (Eca109), glial cell line (HEB), glioma cells (UVW), prostate carcinoma cells (PC-3M, PNT2A, DU145, LNCaP), colon carcinoma cells (CaCO-2, LOVO, COLO-201, HCT-8), cervical carcinoma cells (HeLa), bone osteosarcoma cells (U2-OS), gastric carcinoma cells (AGS), melanoma cells (B16F10, B16F1), ovarian carcinoma cells (OVCAR-3, A2780) and kidney cells (HEK293T). Moreover, it elicited a pronounced activity *in vivo* in skin xenograft and xenograft model of chronic lymphoid leukemia. It exerted its action by targeting several signalling pathways according to the type of cancer, such as reducing the action of topoisomerase 1 and tyrosinase enzyme. Also, it increased the level of p53 expression, induced cell cycle arrest at G1 and or G2/M phase, activated caspase-7 enzyme, elicited apoptosis, and reduced metastasis by inhibiting MMP-2 and -9 (Singh et al., 2020).

Apigenin (17) possesses oral bioavailability of about 30%. Peak plasma concentration of the compound is observed within 0.5–2.5 h post ingestion (DeRango-Adem and Blay, 2021). It is found to be safe, even when administered at elevated doses (Salehi et al., 2019). Reports showed that it elicited *in vitro* cytotoxic activity against various cell lines as mentioned in Supplementary Table S3 (Choi and Kim, 2009b; Souza et al., 2017). Apigenin exerts its activity by different mechanisms such as inducing cell cycle arrest at G2/M phase by decreasing cyclin B1, Cdc2, and Cdc25c expression and by up-regulating p53 and p21 expression. Another mechanism is to induce the intrinsic apoptotic pathway by down-regulating Bcl-2, Bcl-xL, Bcl-w, and Mcl-1 expression and increasing Bad, Bak, Bax, Bid and Bim expression. Also, a decrease in outer mitochondrial membrane potential with cytochrome-c release was observed. Furthermore, it inhibits cell invasion and metastasis through the action on AKT/mTOR pathway. Also, it increases transgelin expression and decreases MMP-9 through the reduction of AKT phosphorylation (Ashrafizadeh et al., 2020). Additionally, an *in vitro* study on MDA-MB-453 cells proved the beneficial effect of combining 5-fluorouracil at its IC_50_ which is equal to 90 µM with apigenin (5, 10, 50, and 100 µM). MDA-MB-453 cells are resistant to 5-fluorouracil due to ErbB2 overexpression. The findings revealed that the proliferation of cancer cells exposed to the combination was highly reduced compared to 5-fluorouracil alone due to decreased resistance owing to the synergistic activity between 5-fluorouracil and apigenin (Choi and Kim, 2009a). In the same context, in an inflammation-induced colon carcinogenesis model, apigenin showed remarkable anticancer effects by reducing myeloperoxidase, inflammatory cytokine, and COX-2 levels and by inhibiting NF-κB and STAT3, thus reducing inflammation. Apigenin was administered at doses of 200 and 300 mg/kg b. w. and exerted its action in a dose dependent manner (Ai et al., 2017). Thus, apigenin is a good candidate for developing chemopreventive and anticancer products. Moreover, a clinical trial carrying the code NCT00609310 and entitled ‘‘Dietary Bioflavonoid Supplementation for the Prevention of Neoplasia Recurrence’’ was carried out to test the consequence of the combination of apigenin with epigallocatechin gallate on the recurrence of colorectal carcinoma. Nevertheless, no results have been released yet (Naponelli et al., 2024).

Astragalin (18) possesses low oral bioavailability and is metabolized by glucuronidation and sulfation. The compound is rapidly eliminated from the body within 1–3 h. Reports showed that its bioavailability and absorption are increased by salt-processing. Moreover, reports revealed that the compound has no toxicity (Chen et al., 2023). Astragalin exerted good *in vitro* and *in vivo* cytotoxic activity against HCT 116 human colon cancer cells. It exhibited an IC_50_ = 18.88 μg/mL. The compound downregulated MMP-2 and MMP-9 expression leading to a decrease in invasion and metastasis. Moreover, it increased caspase-3, -6, -7, -8, and -9 along with p53 and Bax and inhibited Bcl-2, leading to apoptosis. Additionally, it inhibited CDK2, CDK4, cyclin D1, and cyclin E, leading to cell cycle arrest. The *in vivo* study showed that the tumor volume was decreased by 67.06% in the group treated with a dose equal to 75 mg/kg b. w. compared to the untreated control group (Yang et al., 2021). Another study evaluated the cytotoxic activity of astragalin against lung cancer cells. The compound elevated Bax:Bcl-2 ratio and reduced extracellular signal-regulated kinase (ERK)-1/2 and Akt signalling, leading to cell death. Also, it reduced tumor necrosis factor-alpha (TNF*α*)-induced NF-κB activity. Moreover, it enhanced apoptosis in A549 cells when tested at a concentration of 40 μg/mL and also in an *in vivo* animal xenograft model when administered at a dose of 50 mg/kg b. w. (Chen et al., 2017).

Furthermore, it was reported that the combination of two sulphated flavonoids, caicoine (19) and wissadulin (26), exerted cytotoxic activity against PC-3M with IC_50_ (92.14 μg/mL), thus sulphated flavonoids represent an interesting source for developing anticancer drugs; nevertheless, there are no reports about their mechanisms of action (Teles et al., 2015; Teles et al., 2018). Previous reports have shown that chrysin (20) is a good candidate for producing chemopreventive and anticancer agents because it showed promising results in MCF-7 cells by inducing apoptosis. The anti-apoptotic gene c-FLIP-L can stop the activity of caspase-8. Chrysin acts by reducing the activation of NF-κB, leading to the downregulation of the anti-apoptotic NF-κB target gene, which in turn leads to apoptosis. Moreover, it induces caspase-3 leading to AKT or protein kinase B inactivation and to a reduction in the expression of X-linked inhibitor of apoptosis protein (XIAP) (Samarghandian et al., 2011; Samarghandian et al., 2016). Additionally, it exhibited cytotoxic activity against liver cancer cell lines (Huh-7) with IC_50_ = 18.51 μg/mL (El-Din et al., 2014). Moreover, gold nanoparticles synthesized from galangin (21) revealed promising activity against ovarian cancer cells by hastening the p53 level and caspase-8, leading to apoptosis (Al-Shammari et al., 2020).

Reports showed that isoquercetin (22) exerted its cytotoxic activity by reducing the phosphorylation of protein kinase B, enhancing the activity of caspases, and decreasing the levels of anti-apoptotic proteins such as BCl-2 and MCl-1. Also, it scavenges the reactive oxygen species (Akter et al., 2021). Furthermore, reports showed that kaempferol (23) elicited *in vitro* and *in vivo* anticancer activity against breast cancer by down-regulating the expression of cyclin D1, cyclin E, cathepsin D, pIRS-1, pAkt, and pMEK1/2, while up-regulating p21 and Bax expression (Kim et al., 2016). Moreover, it was found that quercetin (24) exerted promising activity *in vitro* against human glioblastoma cells (U251) by reducing the expression of matrix metallopeptidases MMP-9 and MMP-2 (Liu et al., 2017). Also, it was found that it stopped the activity of NF-κB and inhibited the MAPK pathway. Moreover, it inhibited the expression of p-EGFR, VEGFR-2, p-PI3K, Akt, and pGSK3ß; thus, it reduced the rate of carcinogenesis (Pramudya et al., 2022).

Tiliroside (25) is a dietary flavonoid possessing potent antiproliferative activity. However, reports showed that the compound suffers from low oral bioavailability owing to the multidrug resistance-associated protein (MRP2) which affects its intestinal absorption. Therefore, methods should be developed to enhance its bioavailability (Yi et al., 2023). Moreover, the genotoxic and hemolytic potentials of the compound were evaluated using *in vitro* and *ex vivo* methods. The findings proved no cellular toxicity along with low hemolysis rate in RBC (Sousa et al., 2021). It was reported that tiliroside (25) exhibited potent *in vitro* cytotoxic effect against HepG2 cell line with IC_50_ = 3.822 μg/mL which was comparable to the standard drug 5- Fluorouracil (IC_50_ = 0.9 μg/mL). Moreover, the compound was very selective on cancer cell lines (Abdel-Salam et al., 2018). It elicited its action by inhibiting the carbonic anhydrase (CAXII) leading to a decrease in cancer cells growth. Moreover, a decrease in the transcription factors E2F1 and E2F3 expression was observed with an increase in caspase-3 expression, leading to apoptosis (Han et al., 2021). Furthermore, the compound exhibited a cytotoxic activity against T47D cell line by activating caspase-8 and -9 and by decreasing the expression of Bcl-2 protein (Da’i et al., 2016). Therefore, the compound represents a good lead for developing anticancer drugs.

#### 3.2.4 Coumarins

It was reported that scopoletin (27) possessed *in vitro* anticancer activity against human prostate cancer cells by eliciting apoptosis through the induction of caspase-3. Also, it causes cell cycle arrest at G_2_/M phase due to blocking of the PI3K/Akt/mTOR signalling pathway. Moreover, it downregulates the expression of cyclin D1 (Li et al., 2015; Meilawati et al., 2023).

#### 3.2.5 Alkaloids

Alkaloids represent an important class of natural products from which anticancer drugs were developed. It was reported that the anticancer effect of cryptolepine (28) is directly attributed to its anti-inflammatory activity. It was reported that it exerted promising effect against breast cancer due to its effect on cyclin D1, D2, D3 and cyclin E leading to controlling the cell cycle progression. Furthermore, it was reported that it controls IGF-IR expression, thus regulating the PI3k/Akt pathway (Ansha and Mensah, 2013). Moreover, the alkaloid cryptolepinone (29) possessed promising anticancer activity by inducing the quinone reductase activity in mouse hepatoma cells (Hepa 1c1c7) and it also inhibited 7,12-dimethylbenz [*a*]anthracene-induced preneoplastic lesions in mouse mammary organ culture assay (Jang et al., 2003).

#### 3.2.6 Lignans

Reports showed that lignans play crucial role during the early stages of carcinogenesis, so they are regarded as interesting candidates for developing anticancer drugs (Mukhija et al., 2022). Reports showed that boehmenan (30) inhibited the expression of the cytosolic and nuclear *β*-catenin along with *c-myc* in STF/293 cells. Thus, the reduction in *β*-catenin levels reduced the Wnt signal (Shono et al., 2015). Moreover, it was reported that the compounds, namely boehmenan H (31) and *threo*-carolignan K (32) elicited remarkable cytotoxic activity (Pham et al., 2020; Moujir et al., 2007).

#### 3.2.7 Cardiac glycosides

Previous studies showed that the cardiac glycoside glucoevatromonoside (33) exhibited potent anticancer activity against lung cancer with IC_50_ = 19.3 nM compared to the standard drug paclitaxel exhibiting IC_50_ = 260.5 nM. The observed activity of the compound was greater than the standard drug by 14 folds. The compound exerted its action by inducing apoptosis, cell cycle arrest at G2/M phases, and by down-regulating cyclin B1 (Schneider et al., 2018). It was reported that helveticoside (34) represents a promising lead for developing chemopreventive agent against colorectal cancer as it exerted promising *in vitro* effect by increasing Bax level, inhibiting Bcl-2, and by cleaving caspase-3 and -9, leading to apoptosis (An et al., 2020).

#### 3.2.8 Sterols and terpenes

Previous studies showed that betulin (35) and betulinic acid (36) exerted cytotoxic activity against human breast cancer cell lines (MDA-MB231 and HBL-100). They induced apoptosis by increasing the level of apoptotic proteins like Bax, NOXA, PUMA, and PERP and by decreasing the level of anti-apoptotic gene BCl-x. Also, they enhanced cell cycle arrest by activating p21 (Hsu et al., 2015). Moreover, betulinic acid (36) possessed promising anticancer activity by eliciting apoptosis through the activation of mitochondrial apoptotic pathway and the elevation of ROS level. Additionally, it inhibited topoisomerase I, II*α*. Also, it decreased angiogenesis by down-regulating aminopeptidase N (Hordyjewska et al., 2019). Also, it was reported that betulin-3-caffeate (37) exerted cytotoxic activity against human and murine malignant cells (P19, N2/D1, K1735-M2, PC-3, and CaOV3) and A549 cell lines (Shi et al., 2014). Reports showed that *δ*−cadinene (38) elicited cytotoxic activity against MCF-7, BT-20 and HeLa cell lines (Ali et al., 2017; Kubo and Morimitsu, 1995). Furthermore, reports showed that campesterol (39) exerted antiangiogenic activity by reducing the proliferation of endothelial cells and decreasing capillary differentiation (Choi et al., 2007).

In addition, it was previously reported that *β*-caryophyllene (40) exerted *in vitro* cytotoxic activity against various cell lines such as HCT-116, HT-29, and pancreatic cancer cells (PANC-1). It was observed that the compound induced caspase-3, leading to DNA fragmentation. Also, it was found that the compound incorporates itself into the cancer cell membrane, leading to increasing the bilayer membrane permeability, so it helps in enhancing the entrance of the anticancer drug inside the cancerous cell (Fidyt et al., 2016; Dahham et al., 2015).

Furthermore, reports showed that hibiscone C (41) exhibited cytotoxic activity against HeLa cells due to its ability to decrease cell proliferation and to enhance cell death. It was observed that the cell viability was equal to 56.6% when testing hibiscone C at a concentration of 120 µM (Μatsumoto et al., 2020). Moreover, it was observed that hibiscone C reduced the activity of PI_3_K, leading to apoptosis (Besley et al., 2017). Additionally, it was reported that loliolide (42) exerted preventive effect against cell damage induced by H_2_O_2_ (Sebastian et al., 2022; Gangadhar et al., 2020). Moreover, it was reported that palmitic acid (43) elicited pronounced selective cytotoxic activity against MOLT-4. Also, it produced *in vivo* anticancer effect (Thabet et al., 2020). The action of palmitic acid is attributed to its activity on DNA topoisomerase I (Harada et al., 2002).

It was previously reported that phytol (44) possessed anticancer and antimutagenic activities (Sebastian et al., 2022). Reports showed that it exerted effective cytotoxic effect against breast adenocarcinoma MCF-7 and the prostate adenocarcinoma PC-3 cells (Pejin et al., 2014). Moreover, it elicited cytotoxic activity against A549 cell line by elevating the number of cells in the sub-G0 phase, decreasing Bcl-2 level, increasing Bax level, and promoting the activity of caspase-3 and -9. Also, it possessed antiangiogenic activity (Sakthivel et al., 2018).

Moreover, *β*-sitosterol (45) exhibited anticancer and chemopreventive activities against various types of cancer (Singab et al., 2012). An *in vitro* study showed that it exerted antiproliferative effect against MCF-7 cell line and induced apoptosis in MDA-MB 231 cells by activating caspase enzymes (Bin Sayeed and Ameen, 2015). Another *in vitro* study showed that it elicited apoptosis in COLO 320 DM cells by scavenging reactive oxygen species and reducing the expression of *β*-catenin and the PCNA antigens (Baskar et al., 2010). Furthermore, previous studies have shown that it possesses *in vitro* cytotoxic activity against human prostate cancer cell lines by inhibiting cell growth by activating MAPK pathway. Moreover, an *in vitro* study showed that it exerted anticancer effect against SGC-7901 human stomach cancer cells by inducing apoptosis by decreasing the bcl-2/bax ratio and eliciting DNA damage (Bin Sayeed and Ameen, 2015). In the same context, it was reported that γ-sitosterol (46) exhibited its cytotoxic activity by activating the apoptotic pathway by decreasing *c-myc* oncogene expression (Sebastian et al., 2022; Endrini et al., 2014).

*In vitro* studies have shown that stigmasterol (47) exerts promising activity against the human gastric cancer cell line SNU-1 by eliciting antiproliferative effects, inducing apoptosis, and exhibiting cell cycle arrest at G2/M phases (Li et al., 2018). Furthermore, stigmasterol has been reported to decrease capillary formation and reduce endothelial cell proliferation and migration; therefore, it elicits antiangiogenic activity. Also, it exhibited antiproliferative activity against HepG2 and MCF-7 cells. Moreover, it increased the levels of Bax, caspase-3, and -9 and decreased Bcl-2 expression (Kadhum et al., 2021; Al-Fatlawi, 2019).

Furthermore, it was reported that tanshindiol (48) elicited anticancer effect through reducing the activity of EZH2 (Woo et al., 2014). It was previously reported that ursolic acid (49) elicited promising *in vitro* cytotoxic activity against breast cancer cells (MCF-7) by inducing apoptosis and by increasing ROS production (Mishra et al., 2016). Another *in vitro* study showed that it possessed significant anticancer activity against prostate cancer by inducing apoptosis and by down-regulating Bcl-2 protein (Kassi et al., 2007).

#### 3.2.9 Polysaccharides

Previous reports showed that polysaccharides elicited promising anticancer activity due to their ability to scavenge radicals, inhibit DNA topoisomerase, induce apoptosis, and prevent angiogenesis (Yarley et al., 2021). It was found that glucans (50) possessed potent antioxidant and chemopreventive activities (Oliveira et al., 2013). Furthermore, it was reported that pectin (51) gold nanoparticles enhanced apoptosis and DNA damage in several types of cancer like mammary adenocarcinoma cell lines (Suganya et al., 2016). Also, it possessed antioxidant and anti-inflammatory activities (Yan et al., 2023; Teng et al., 2022).

### 3.3 *In silico* pharmacokinetic profile, oral bioavailability, drug likeness and acute oral toxicity study of the bioactive cytotoxic compounds and the reference drug erlotinib

**FIGURE 2:**
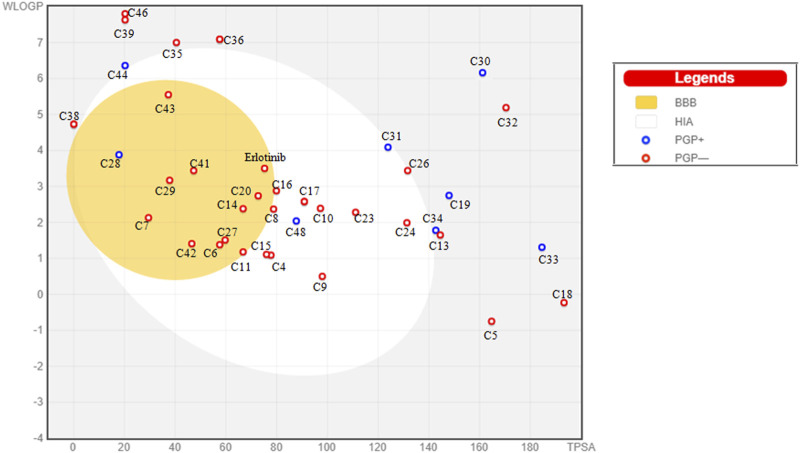
BOILED-Egg chart revealing the predicted absorption of the reported cytotoxic metabolites and erlotinib.

The results revealed that the compounds 4, 6, 7, 8, 9, 10, 11, 14, 15, 16, 17, 20, 21, 23, 24, 26, 27, 28, 29, 31, 41, 42, 43, 48, and erlotinib possessed features for high gastrointestinal absorption owing to their observed solubility. Moreover, compounds 6, 7, 11, 14, 20, 27, 28, 29, 41, 42, 43, and erlotinib have abilities for blood brain barrier permeability. In addition, compounds 8, 9, 10, 14, 16, 17, 20, 21, 23, 24, 28, 29, 31, and erlotinib showed effect on CYP 3A4, while other compounds were devoid of this effect; thus, preventing any possible drug-drug interactions. The results are listed in Table 1 and illustrated in Figure 2. Moreover, the drug likeness properties of the tested compounds were assessed using the Lipinski’s rule-of-5 (Table 1). A compound having a molecular weight below 500 g/mol, no more than 10 hydrogen bond acceptors, less than 5 hydrogen bond donors and a Log P below 5 and that violates no more than one of the stated criteria, is considered to obey the Lipinski’s rule-of-5 (Ivanović et al., 2020). The findings of the study showed that compounds 12, 18, 22, 25, 30, 32, 33, 37, 50, and 51 elicited more than one violation to the Lipinski’s rule-of-5. Additionally, the Bioavailability Radar map illustrated the drug likeness features and the bioavailability of each compound (Supplementary Table S4). Furthermore, the predicted acute oral toxicity (LD_50_) and the toxicity class of each compound were determined using Pro Tox 3.0. The acute oral toxicity is classified into 6 classes as follows: class 1 with LD_50_ ≤ 5 mg/kg and class 2 having LD_50_ more than 5 and less than or equal to 50 mg/kg are considered to be fatal if swallowed, class 3 possessing LD_50_ more than 50 and less than or equal to 300 mg/kg is found to be toxic if swallowed, class 4 with LD_50_ more than 300 and less than or equal to 2000 mg/kg is harmful if swallowed, class 5 possessing LD_50_ more than 2000 and less than or equal to 5,000 mg/kg may be harmful if swallowed and class 6 with LD_50_ > 5,000 mg/kg is nontoxic (Banerjee et al., 2024). Compounds 33, 41, and 42 were predicted to be fatal, while the compounds 29, 50, 51, and erlotinib were found to be toxic. The results are listed in Table 1.

**TABLE 1:** Predicted pharmacokinetic profile, drug-likeness and acute oral toxicity of the bioactive cytotoxic metabolites and erlotinib.

Compound	TPSA	HBA	HBD	Log P	M_W_	Solubility	GI absorption	BBB permeability	CYP 3A4 inhibition	Lipinski’s violation	LD_50_ (mg/kg)	Acute oral toxicity class
1	0.00	0	0	5.93	536.87	Poorly soluble	Low	No	No	1	1,510	4
2	0.00	0	0	5.93	536.87	Poorly soluble	Low	No	No	1	5,700	6
3	40.46	2	2	5.93	568.88	Poorly soluble	Low	No	No	1	10	2
4	77.76	4	3	0.97	180.16	Very soluble	High	No	No	0	2,980	5
5	164.75	9	6	0.87	354.31	Very soluble	Low	No	No	1	5,000	5
6	57.53	3	2	0.95	164.16	Soluble	High	Yes	No	0	2,850	5
7	29.46	2	1	2.37	164.20	Soluble	High	Yes	No	0	1930	4
8	78.79	4	3	2.39	313.35	Soluble	High	No	Yes	0	500	4
9	97.99	5	4	0.21	170.12	Very soluble	High	No	Yes	0	2000	4
10	97.25	6	3	3.14	373.40	Soluble	High	No	Yes	0	500	4
11	66.76	4	2	1.81	194.18	Soluble	High	Yes	No	0	2,980	5
12	246.8	15	7	2.79	692.62	Moderately soluble	Low	No	No	3	5,000	5
13	144.52	8	5	1.48	360.31	Soluble	Low	No	No	0	5,000	5
14	66.76	4	2	2.21	232.23	Soluble	High	Yes	Yes	0	1,330	4
15	75.99	5	2	1.54	198.17	Very soluble	High	No	No	0	1700	4
16	79.90	5	2	2.56	284.26	Moderately soluble	High	No	Yes	0	4,000	5
17	90.90	5	3	1.89	270.24	Soluble	High	No	Yes	0	2,500	5
18	190.28	11	7	−0.09	448.38	Soluble	Low	No	No	2	5,000	5
19	147.97	9	3	1.66	382.34	Soluble	Low	No	No	0	2000	4
20	70.67	4	2	2.55	254.24	Moderately soluble	High	Yes	Yes	0	3,919	5
21	90.90	5	3	2.08	270.24	Soluble	High	No	Yes	0	3,919	5
22	210.51	12	8	0.94	464.38	Soluble	Low	No	No	2	5,000	5
23	111.13	6	4	1.70	286.24	Soluble	High	No	Yes	0	3,919	5
24	131.36	7	5	1.63	302.24	Soluble	High	No	Yes	0	159	3
25	216.58	13	7	2.68	594.52	Moderately soluble	Low	No	No	3	5,000	5
26	131.65	8	2	1.82	364.33	Moderately soluble	High	No	No	0	4,000	5
27	59.67	4	1	1.86	192.17	Soluble	High	Yes	No	0	3,800	5
28	17.82	1	0	2.46	232.28	Moderately soluble	High	Yes	Yes	0	2000	4
29	37.79	1	1	2.15	248.28	Moderately soluble	High	Yes	Yes	0	300	3
30	159.44	12	3	2.36	712.74	Poorly soluble	Low	No	No	2	1,000	4
31	123.91	9	3	4.76	536.57	Moderately soluble	High	No	Yes	1	5,000	5
32	170.44	12	4	5.59	698.71	Poorly soluble	Low	No	No	2	5,000	5
33	184.60	12	6	4.40	666.80	Moderately soluble	Low	No	No	3	4	1
34	142.75	9	4	2.52	534.64	Soluble	Low	No	No	1	2000	4
35	40.46	2	2	6.39	442.72	Poorly soluble	Low	No	No	1	2000	4
36	57.53	3	2	6.14	456.70	Poorly soluble	Low	No	No	1	2,610	5
37	86.99	5	3	7.45	604.87	Poorly soluble	Low	No	No	2	9,600	6
38	0.00	0	0	3.41	204.35	Soluble	Low	No	No	0	4,390	5
39	20.23	1	1	6.92	400.68	Poorly soluble	Low	No	No	1	890	4
40	0.00	0	0	3.25	204.35	Soluble	Low	No	No	0	5,300	5
41	47.28	3	0	2.27	246.30	Soluble	High	Yes	No	0	38	2
42	46.53	3	1	2.01	196.24	Very soluble	High	Yes	No	0	34	2
43	37.30	2	1	3.85	256.42	Moderately soluble	High	Yes	No	0	900	4
44	20.23	1	1	6.25	296.53	Moderately soluble	Low	No	No	1	5,000	5
45	20.23	1	1	7.19	414.71	Poorly soluble	Low	No	No	1	890	4
46	20.23	1	1	7.19	414.71	Poorly soluble	Low	No	No	1	890	4
47	20.23	1	1	5.08	412.69	Poorly soluble	Low	No	No	1	890	4
48	87.74	5	2	2.20	312.32	Soluble	High	No	No	0	1,230	4
49	57.53	3	2	5.93	456.70	Poorly soluble	Low	No	No	1	2000	4
50	268.68	16	11	−1.84	504.44	Highly soluble	Low	No	No	3	51	3
51	229.33	13	8	−4.52	384.1	Highly soluble	Low	No	No	2	90	3
Erlotinib	74.73	6	1	3.20	393.44	Moderately soluble	High	Yes	Yes	0	125	3

TPSA: topological polar surface area, HBA: hydrogen bond acceptors, HBD: hydrogen bond donors, Log P: logarithm of the Partition Coefficient, MW: molecular weight, GI: gastrointestinal, BBB: blood brain barrier, CYP, 3A4: Cytochrome P450 3A4, LD50: Lethal dose, 50%.

### 3.4 *In silico* molecular modelling of the reported bioactive cytotoxic compounds

**FIGURE 3:**
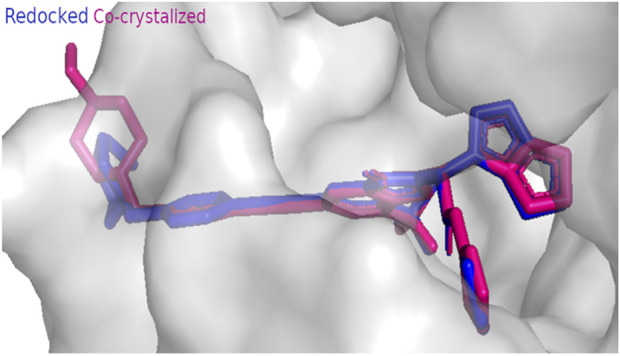
Docking protocol validation result of EGFR kinase showing RMSD value equal to 0.414 A° (co-crystallized pose = pink, docked pose = blue).

**FIGURE 4:**
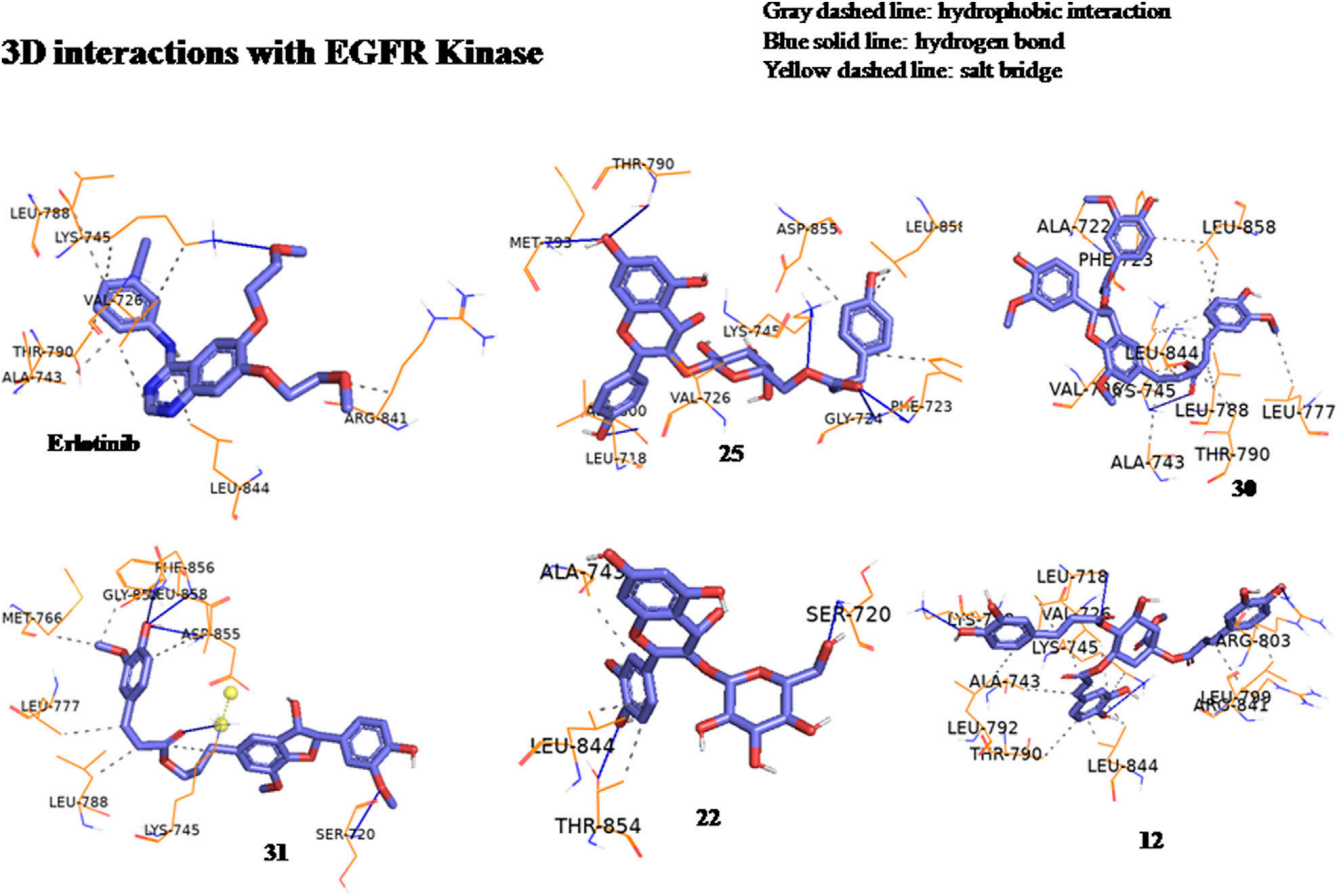
Three-dimensional interactions of erlotinib and compounds tiliroside (25), boehmenan (30), boehmenan H (31), isoquercetin (22) and methyl-1,4,5-tri-*O*-caffeoyl quinate (12) with EGFR kinase (8A27) active sites.

**FIGURE 5:**
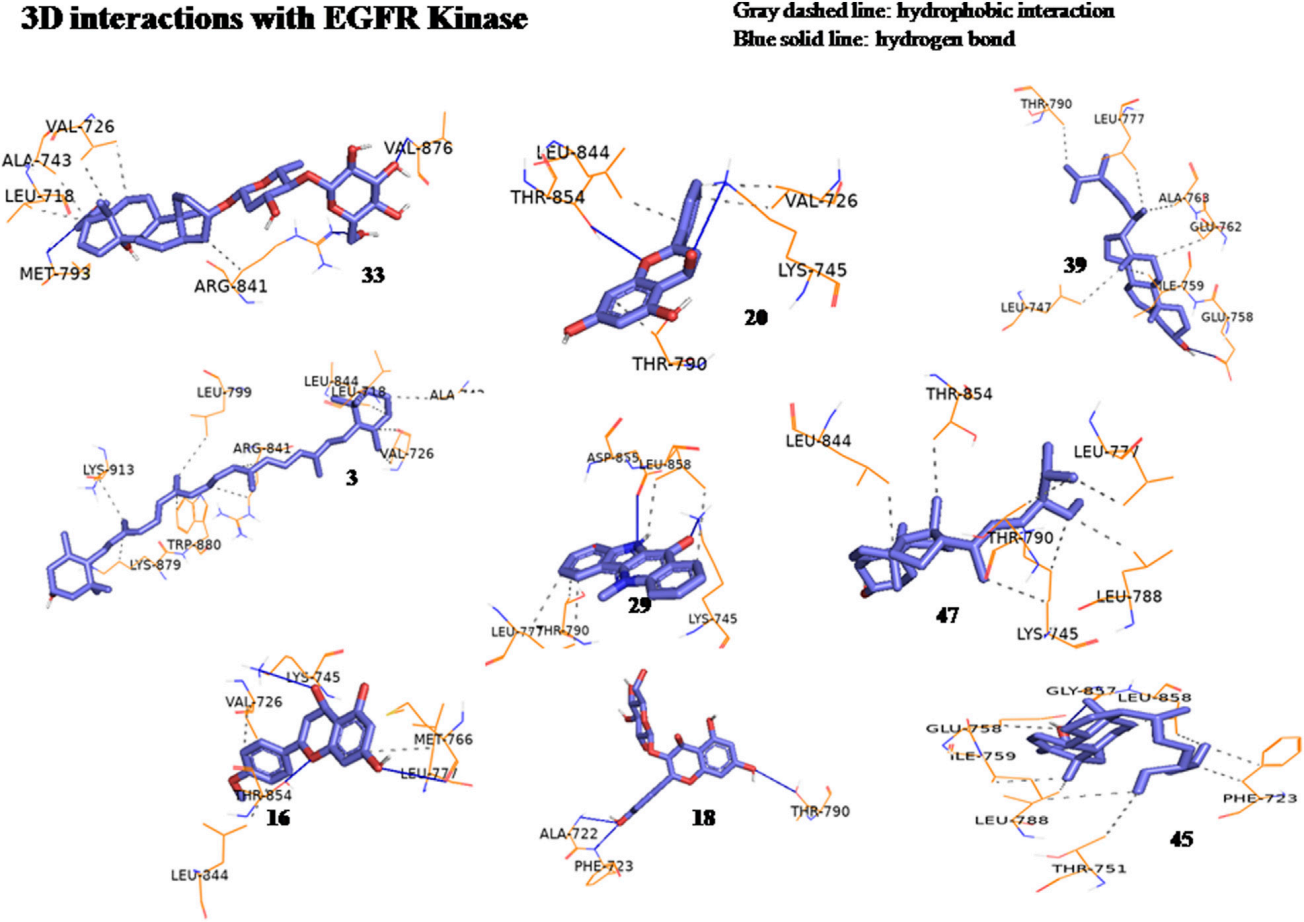
Three-dimensional interactions of compounds glucoevatromonoside (33), chrysin (20), campesterol (39), zeaxanthin (3), cryptolepinone (29), stigmasterol (47), acacetin (16), astragalin (18) and *β*-Sitosterol (45) with EGFR kinase (8A27) active sites.

The anticancer effect of the reported compounds was studied through testing their inhibitory potential against EGFR kinase enzyme. Erlotinib was used as the reference anticancer drug as it could arrest the EGFR pathway. The docking protocol was validated (Figure 3). Results revealed that all the screened compounds belonging to different classes of bioactive metabolites possessed agreeable binding affinities with negative binding scores to the EGFR kinase enzyme compared to the reference drug erlotinib which elicited a binding score equal to −9 kcal/mol. Furthermore, it was observed that compounds 25, 30, 31, 22, 12, 33, 20, 39, 3, 29, 47, 16, 18, and 45 elicited binding affinities higher than the reference drug with scores equal to −10.4, −10.4, −10.2, −10.1, −9.7, −9.6, −9.5, −9.4, −9.3, −9.3, −9.3, −9.1, −9.1 and −9.1 kcal/mol, respectively, due to the observed hydrophobic interactions and hydrogen bonds between the compounds and the receptor. The binding scores of all the tested compounds are listed in Table 2. The binding interactions of the compounds with the highest affinities to the enzyme are listed in Supplementary Table S5 and shown in Figures 4, 5.

**TABLE 2:** Docking scores of the reported bioactive cytotoxic metabolites with EGFR kinase (8A27).

	Score (Kcal/mol)
1	−8.1
2	−8.4
3	−9.3
4	−6.7
5	−6.6
6	−6.5
7	−5.9
8	−8.9
9	−6.3
10	−8.9
11	−6.6
12	−9.7
13	−7.1
14	−6.6
15	−6
16	−9.1
17	−8.9
18	−9.1
19	−8
20	−9.5
21	−8.2
22	−10.1
23	−7.6
24	−8.6
25	−10.4
26	−8.7
27	−6.9
28	−8.5
29	−9.3
30	−10.4
31	−10.2
32	−8.8
33	−9.6
34	−8.7
35	−8.2
36	−8
37	−8.2
38	−7.1
39	−9.4
41	−7.9
42	−7.2
43	−6.3
44	−7.3
45	−9.1
47	−9.3
48	−8.9
49	−8.3
50	−7
51	−7.1
Erlotinib	−9

### 3.5 *In silico* molecular dynamic simulations and MM-GBSA calculations

**FIGURE 6:**
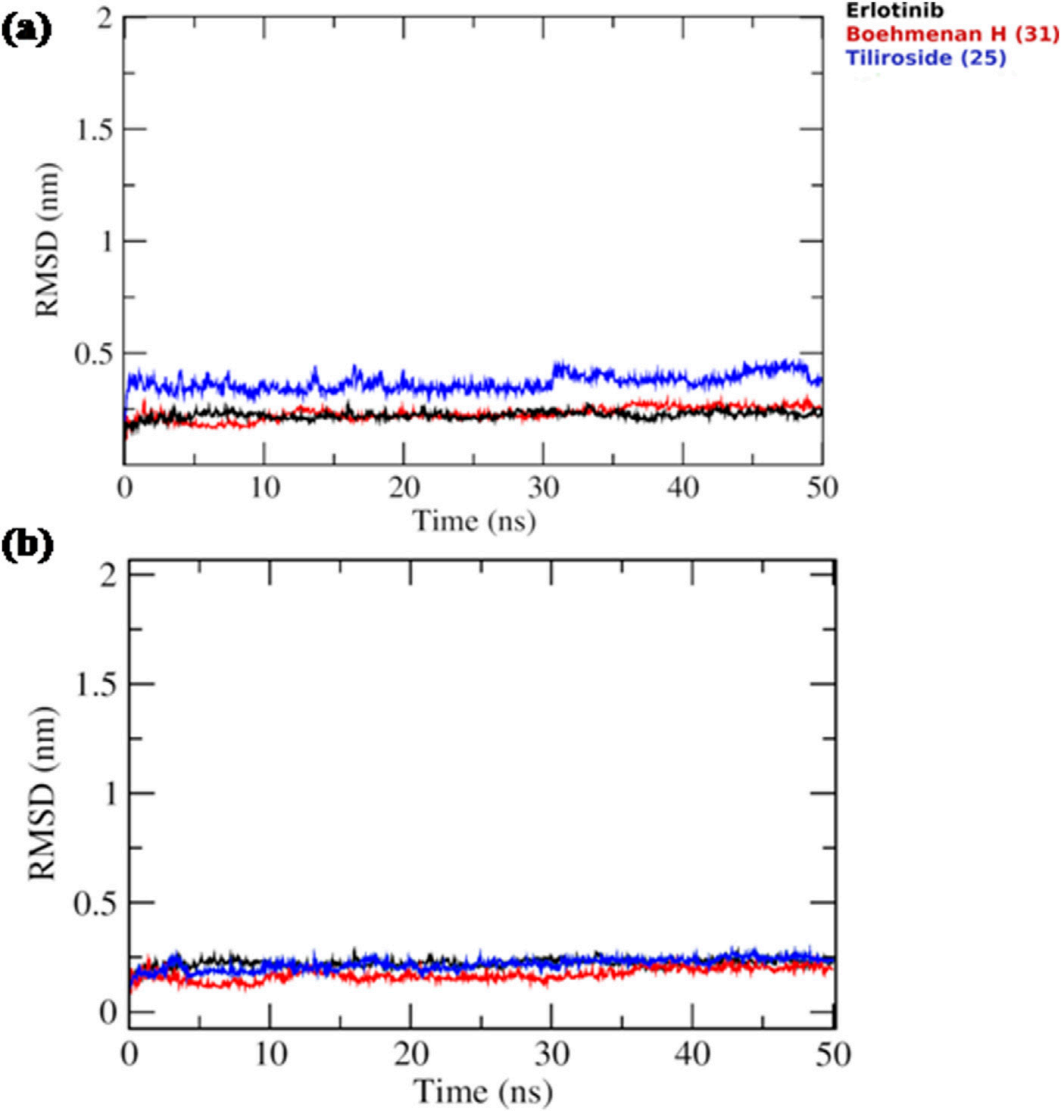
**(A)** Ligand RMSD for boehmenan H (31), tiliroside (25), and erlotinib. **(B)** Protein RMSD of EGFR.

**FIGURE 7:**
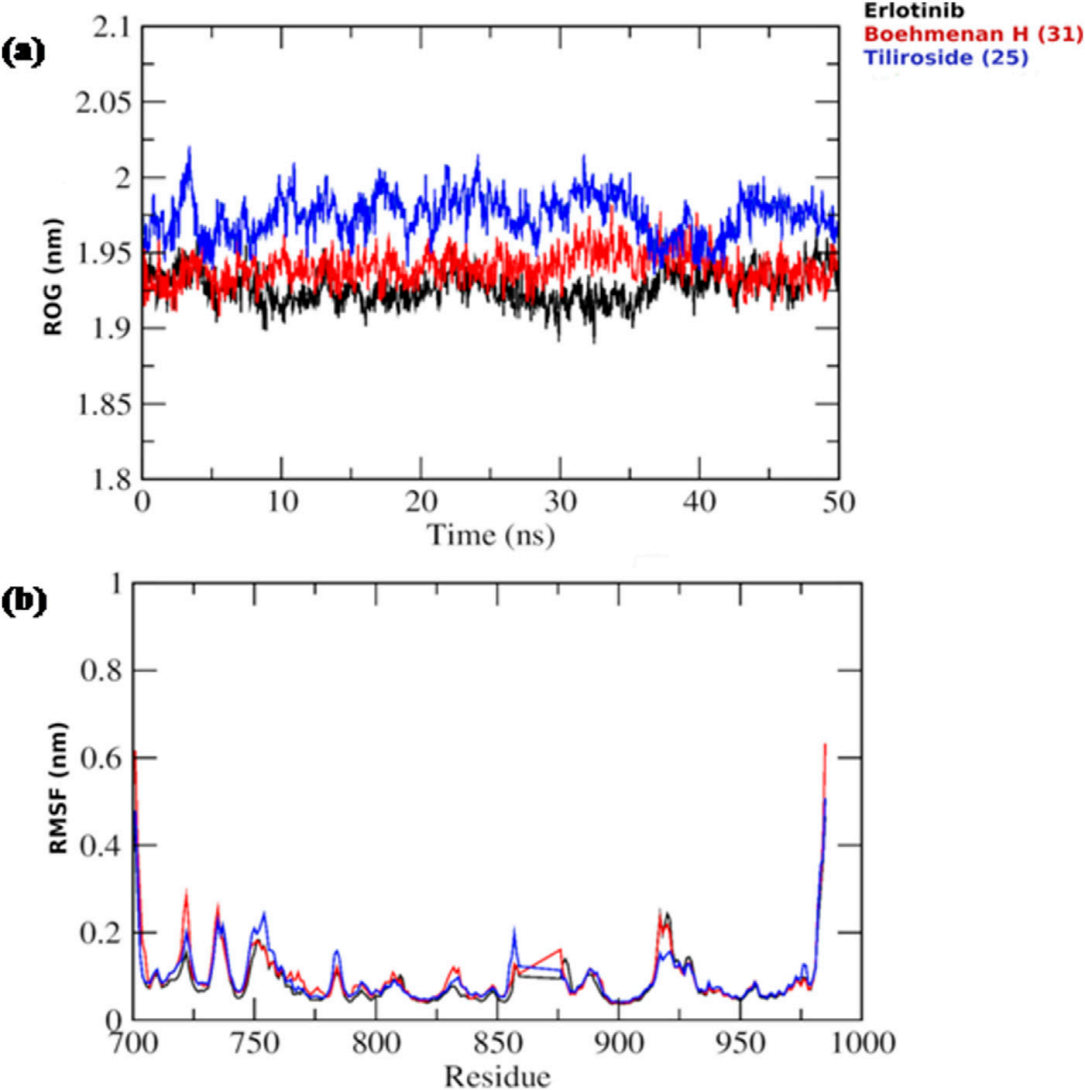
**(A)** Radius of gyration (RoG) of the EGFR throughout the simulation time for boehmenan H (31), tiliroside (25) and erlotinib. **(B)** Per residue root mean square fluctuation (RMSF) with the amino acid residues of the binding site are presented.

Based on the docking results, it was observed that tiliroside (25) and boehmenan H (31) exhibited high binding affinities toward EGFR kinase comparable to erlotinib. In addition, previous reports revealed the potent *in vitro* cytotoxic activity of the compounds. Therefore, molecular dynamic simulations were performed for 50 ns to evaluate the binding modes and stability of the candidate compounds under real physiological conditions. The RMSD value estimated the stability of the ligand-protein complexes based on these simulations. EGFR backbone equilibrated at below 0.5 nm for every complex as shown in Figure 6B. The simulations started with a lower RMSD value for the boehmenan H (31) complex and remained consistent by the end of the simulation. Erlotinib and tiliroside (25) exhibited comparable effect on EGFR stability (Figure 6B). The RMSD of boehmenan H (31) and erlotinib seemed to overlap at the lower value, suggesting that they had a more solid docking position than the other compound. However, compound tiliroside (25) fluctuated especially after 30 ns, but maintained below 0.5 nm (Figure 6A). The conformational variations per residue during the period of the simulation were measured by root mean square fluctuation (RMSF). The two ligands possessed comparable patterns of flexibility. However, erlotinib showed more stable fluctuation. In contrast, all of them had variation less than 0.4 nm, except for residues around720 and 970 (Figure7B). Acceptable radius of gyration (RoG) ranges were shown by the complex systems. The erlotinib system (black) and boehmenan H (31) (red) elicited RoG values below 2 nm. Tiliroside (25) fluctuated higher than 2 nm but lower than 2.05 nm (Figure 7A).

**FIGURE 8:**
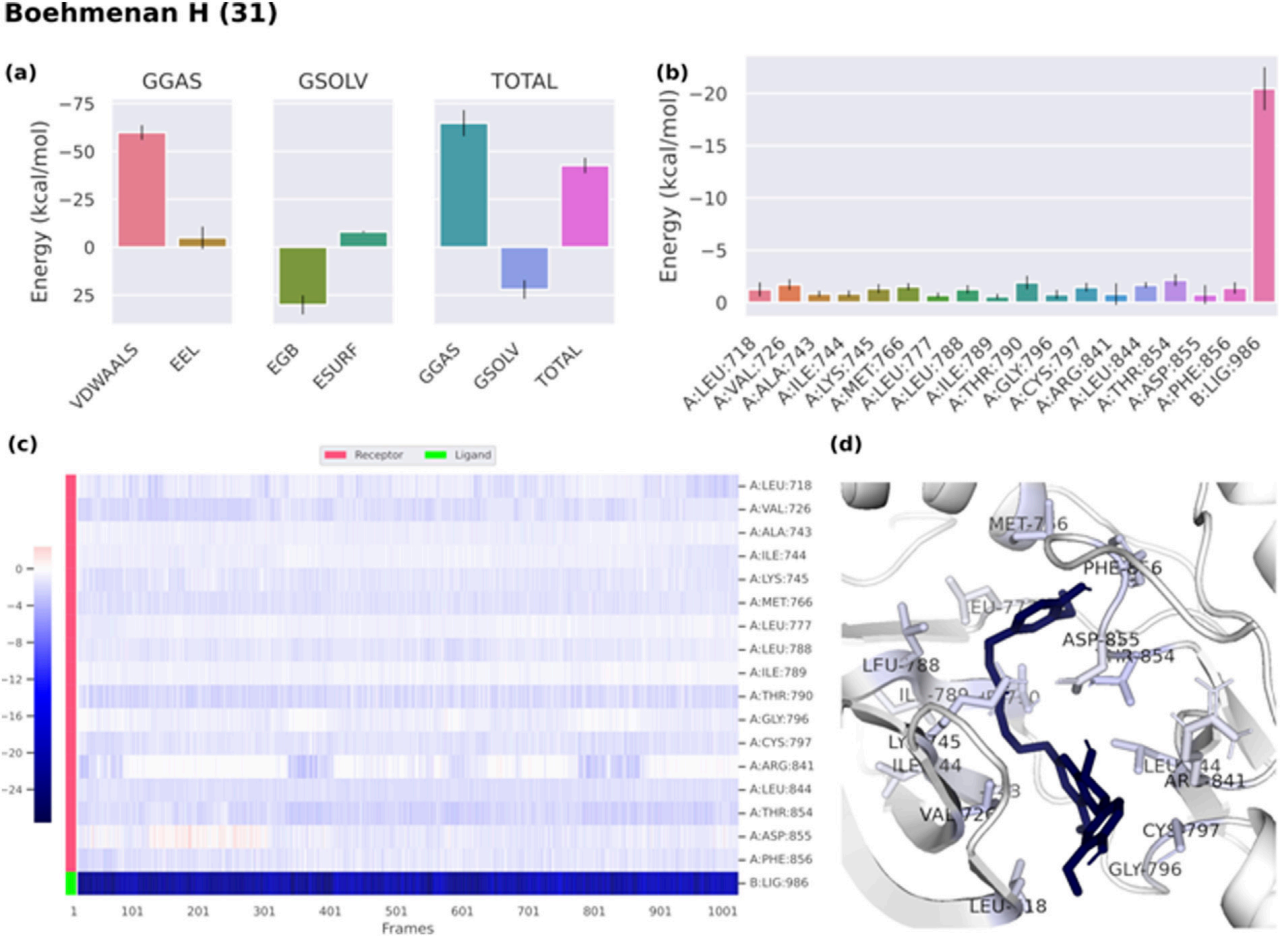
**(A)** Free energy components of boehmenan H (31). **(B)** Energy contribution of each individual residue of EGFR. **(C)** Heatmap displaying energy contribution of each residue against time. **(D)** 3D illustration of the binding.

**FIGURE 9:**
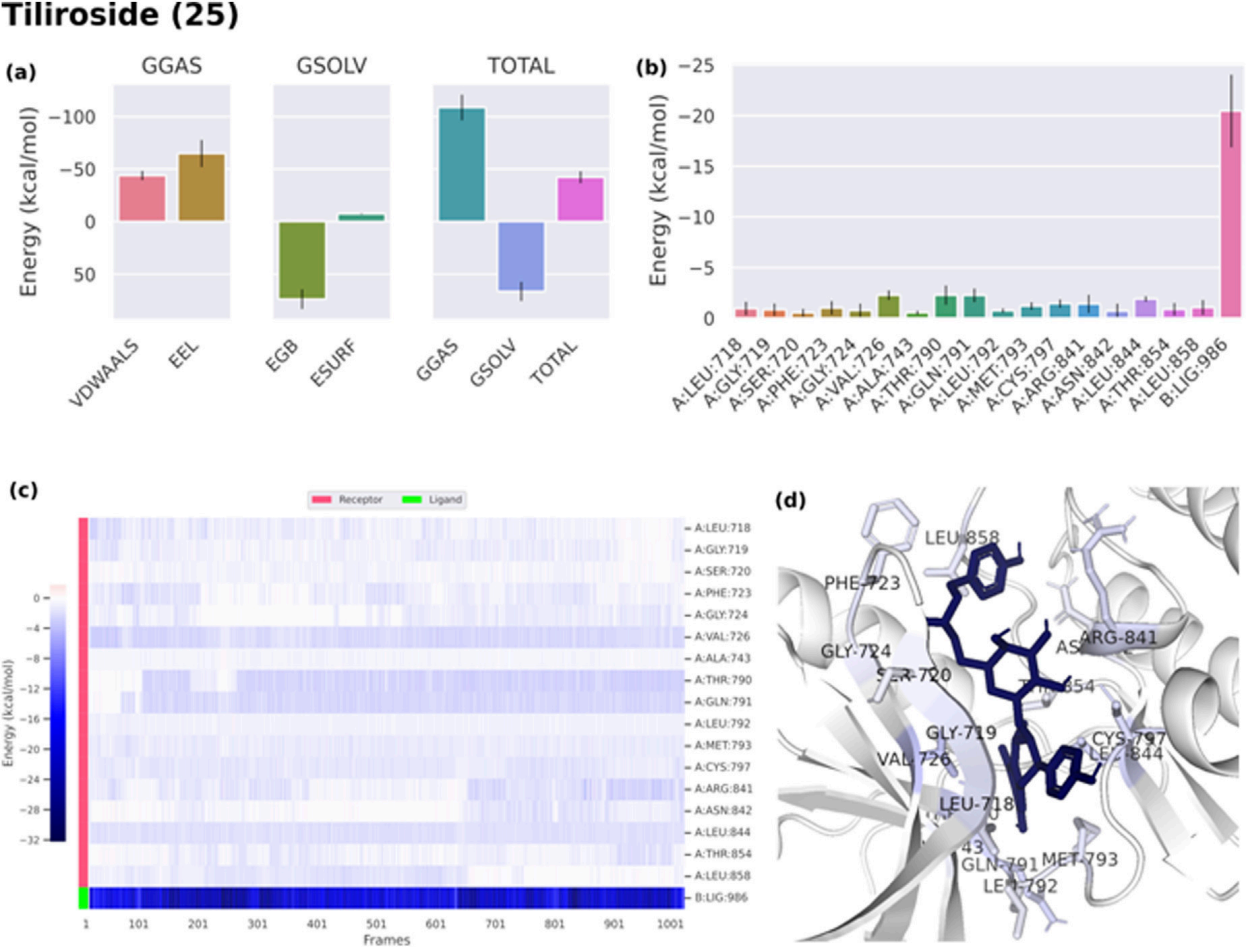
**(A)** Free energy components of tiliroside (25). **(B)** Energy contribution of each individual residue of EGFR. **(C)** Heatmap displaying the energy contribution of each residue against time. **(D)** 3D illustration of the binding.

**FIGURE 10:**
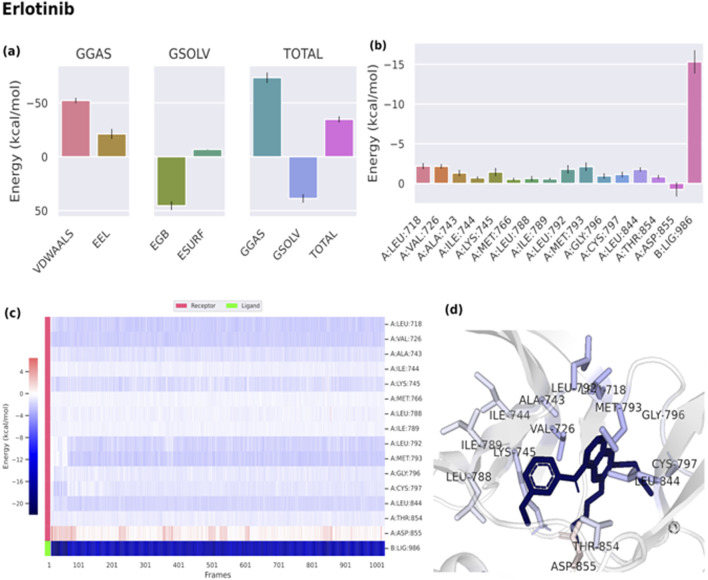
**(A)** Free energy components of erlotinib. **(B)** Energy contribution of each individual residue of EGFR. **(C)** Heatmap displaying the energy contribution of each residue against time. **(D)** 3D representation of the binding.

Binding free energies (ΔG) were computed and averaged for each of the three complexes over the course of the 50 ns simulations for comparative purpose as it gives useful information for estimating the total binding strength. For all complex systems, the binding free energy (ΔG) and its components are shown in Table 3. Gibbs free energy of the gas (ΔG_GAS_) and the solvation free energy (G_solv_) were employed to compute the free energies. ΔG_GAS_ is the sum of electrostatic energy (Δ_ELE_) and van der Waals energy (VDWAALS) (Genheden and Ryde, 2015). G_solv_ is the sum of the non-polar solvation energies Δ_ESURF_, and the generalized Born Polar solvation energy (E_GB_). The total energy components for the binders were plotted as shown in Figures 8A, 9A for compounds 31 and 25, respectively. The energy composition analysis verified the strong interaction of compound (31) with Val726, Thr854, Thr790, Lys745, Phe856, Met766, Leu844 of the EGFR, as displayed in the individual energy contribution graph (Figure 8B). Also, the heatmap and 3D interactions highlighted some residues to maintain the individual energy contribution throughout the 50 ns simulation’s time, especially, Thr790 and Met766 (Figures 8C, D). Regarding tiliroside (25), the individual energy contribution graph revealed favorable interactions with the previous amino acids, but the most contributing residues were Thr790, and Gln791 (Figure 9B). Notably, the heatmap and 3D interactions (Figures 9C, D) confirmed the maintenance of the energy contribution through the simulation’s time. The obtained results were comparable to the standard drug erlotinib (Figure 10).

**TABLE 3:** Binding free energy of ligand-EGFR complexes.

	Energy (Kcal/mol)						
	VDWAALS	Δ_ELE_	ΔE_GB_	ΔE_SURF_	ΔG_GAS_	ΔG_SOLV_	ΔTOTAL
Boehmenan H (31)	−59.87	−15.57	30.12	−8.01	−64.79	22.11	−42.68
Tiliroside (25)	−21.18	−64.79	73.79	−7.35	−108.61	66.44	−42.17
Erlotinib (Standard)	−52.2	−38.21	45.44	−6.75	−73.37	38.68	−34.69

## 4 Discussion

Current studies showed that phytochemicals could be employed as alternative or complementary routes for alleviating cancer by re-establishing the normal epigenetic marks which are changed due to tumourigenesis. Herbal nutraceuticals are considered to be dietary supplements exhibiting potent health benefits and could be employed in the prevention and treatment of cancer as the bioactive phytochemicals are able to decrease the growth and proliferation of tumor cells (Calvani et al., 2020).

By reviewing the literature, it was observed that some members belonging to family Malvaceae were utilized in folk medicine to relieve tumors. Moreover, reports showed that family Malvaceae members are rich with potent cytotoxic compounds belonging to several phytochemical classes, such as carotenoids, phenolic acids, flavonoids, coumarins, alkaloids, lignans, cardiac glycosides, sterols, terpenes and polysaccharides, which represent good sources of lead compounds possessing chemopreventive and anticancer activities. However, scarce data have summarized the cytotoxic activity and the anticancer mechanisms of the bioactive compounds in the Malvaceae family. The study will draw the attention to medicinal plants belonging to family Malvaceae that possess anticancer activity and the possible anticancer mechanisms of action of the cytotoxic compounds. The search results revealed that the cytotoxic compounds were subjected to *in vitro* and *in vivo* studies with limited clinical trials. Clinical trials represent a challenge in the production of natural anticancer drugs because of difficulties in designing and recruiting participants (Chunarkar-Patil et al., 2024).

Additionally, the current study provides valuable insights on the pharmacokinetic and pharmacodynamic profiles of the reported cytotoxic compounds. Recently, it was observed that computational modelling including ADME prediction and molecular docking, exerts a pivotal role in the discovery of anticancer lead compounds. This approach increases the rate of anticancer drug discovery and reduces the cost needed for developing chemopreventive and anticancer medications. Despite the importance of the computational approach, validation of the results through experimental procedures such as preclinical and clinical studies remains crucial to verify the effectiveness and safety of the tested natural compounds (Chunarkar-Patil et al., 2024). Errors may exist in the pharmacokinetic estimates generated by computational tools, which could affect the activity of drug candidates during clinical trials (Mushebenge et al., 2023). It is important to note that several natural products exhibit potential activity in the *in vitro* and *in vivo* studies, while their effects vary in clinical trial inside the human body owing to their poor bioavailability (Phansalkar et al., 2020). In case of oral drugs, gastrointestinal absorption represents an obstacle between the bioactive ingredients and the blood circulation. Therefore, the bioavailability of natural entities affects the process of oral drug development. A drug that obey Lipinski’s rule-of-five is considered to possess favorable features for gastrointestinal absorption (Abourashed, 2013). However, the removal of natural compounds that violate the rule-of-five from further studies, could conduct to missing promising effective drugs. An equilibrium between efficacy and safety should be taken in consideration during the complex process of drug development (Ivanović et al., 2020; Beutler, 2009; Li et al., 2024). Notably, flavonoids possess a wide range of pharmacological applications; however, reports showed that they elicit low oral bioavailability owing to their low aqueous solubility, which in turn remarkably affects their efficacy. Several strategies have been developed to improve oral bioavailability such as the employment of absorption enhancer, structural transformation. In addition, carrier complexes, nanotechnology and co-crystals represent promising technologies (Zhao et al., 2019).

Preliminary pharmacokinetic prediction represents a crucial step in drug discovery (Phansalkar et al., 2020). In the present study, the white region of the BOILED-Egg chart (Figure 2) revealed that compounds 4, 9, 10, 15, 17, 23, 24, 31, and 48 possessed high probability of passive gastrointestinal absorption (HIA). Moreover, the predicted effect of the compounds on CYP3A4 was studied. CYP3A4 represents 30% of the total P450 quantity in the liver. Many anticancer medications are metabolized by CYP3A4. Patients suffering from cancer are administered combination remedy which may lead to possible drug-drug interactions, severe side effects, serious toxicities or could reduce drug’s effectiveness (Ando, 2004; Tian and Hu, 2014). The tested compounds 4, 5, 6, 7, 11, 12, 13, 15, 18, 19, 22, 25, 26, 27, 30, 32, 33, 34, 35, 36, 37, 38, 39, 40, 41, 42, 43, 44, 45, 46, 47, 48, 49, 50, and 51 showed no effect on CYP3A4 eliminating any possible interactions or side effects as mentioned in Table 1.

Furthermore, P-glycoprotein represents a major challenge facing the development of successful chemotherapeutic agents. P-glycoprotein is responsible for the development of multidrug resistance. It is highly expressed in blood cancers and solid tumors leading to poor clinical outcomes (Waghray and Zhang, 2017). By observing the Boiled-Egg chart (Figure 2), it was found that compounds 4, 5, 6, 7, 8, 10, 11, 13, 14, 15, 16, 17, 18, 23, 24, 26, 29, 32, 35, 36, 38, 39, 41, 42, 43, 46, and erlotinib are illustrated in red color showing that they are non-substrate of P-gp (PGP^−^).

The Bioavailability Radar map generated using SWISS ADME revealed the drug likeness and the oral bioavailability features of the reported compounds. It was observed that compounds 5, 12, 18, 22, 25, 30, 32, 33, 37, 50, and 51 possessed a poor bioavailability score, while the other compounds showed good oral bioavailability profile. Moreover, toxicity studies should be carried out on natural substances during drug development (Dzobo, 2022). The acute oral toxicity profile of the cytotoxic metabolites was predicted using Pro Tox 3.0.

The *in silico* molecular docking study was performed on the reported cytotoxic metabolites against EGFR kinase PDB ID (8A27). The epidermal growth factor receptor (EGFR) belongs to tyrosine kinase family and is responsible for tumor growth and metastasis; therefore, it represents a potential therapeutic target (Sasaki et al., 2013). Erlotinib was used as the reference drug as it possessed potent EGFR kinase inhibitory activity (Ling et al., 2007). The results revealed that all the tested compounds revealed agreeable binding affinities toward the enzyme with negative binding scores owing to the hydrophobic interactions and the hydrogen bonds between the compounds and the amino acid residues present in the receptor. It is important to note that hydrogen bonding affects the compound solubility, distribution and permeability. Also, it makes the binding between the drug and receptor easier. Also, hydrophobic interactions play a pivotal role in establishing the binding affinity between the compound and the receptor and determine the selectivity of the drugs toward the target (Afolabi et al., 2024).

Natural products exert a pivotal role in anticancer drug discovery. Drug discovery is a multifactorial approach which requires a balance between the pharmacodynamic and pharmacokinetic profiles of the compounds in order to select the best drug candidates (Guan et al., 2019). The current study revealed that several reported cytotoxic metabolites possessed good pharmacokinetic features and elicited high binding affinity against EGFR kinase enzyme. It is important to note that compounds 25, 30, 31, 22, 12, 33, 20, 39, 3, 29, 47, 16, and 45 showed potent inhibitory activity toward EGFR kinase receptor. In addition, by performing molecular dynamic simulations studies on compounds 25 and 31, it was observed that they possessed a good stability in the receptor comparable to the standard drug erlotinib. Further in-depth preclinical and clinical studies should be carried out to develop safe and effective chemopreventive and anticancer medications. The present study represents a point of departure toward the selection of good candidates from family Malvaceae to be a nucleus for future plant based anticancer drug development.

## 5 Conclusion

Family Malvaceae represents a promising source of bioactive phytometabolites belonging to various classes, such as carotenoids, phenolic acids, flavonoids, coumarins, alkaloids, lignans, cardiac glycosides, sterols, terpenes, and polysaccharides. By reviewing the literature, we observed that some members were utilized traditionally for curing tumors. Moreover, cytotoxic studies showed that many species possessed promising chemopreventive and anticancer activities. Nevertheless, data about the possible mechanisms of action are scarce. Furthermore, a very small number of isolated compounds have been subjected to cytotoxic studies. Natural products are characterized by their biofunctionality and biodiversity and will represent a revolution in cancer management in the upcoming decade owing to their biodegradability and biocompatibility. Drug discovery from natural products is a complex process which faces many obstacles such as absence of enough studies concerning the efficiency, safety, stability and favorable drug targets. This study is an endeavor to summarize the reported anticancer activity of species of family Malvaceae and to provide pharmacokinetic and pharmacodynamic profiles for the bioactive cytotoxic compounds. The results of the *in silico* study showed that the reported bioactive metabolites exhibited good binding affinities to the EGFR kinase enzyme. The compounds, namely tiliroside (25), boehmenan (30), boehmenan H (31), and isoquercetin (22) revealed safe pharmacokinetic properties. They elicited the highest binding affinity toward EGFR kinase enzyme with a score of −10.4, −10.4, −10.2 and −10.1 Kcal/mol, respectively, in comparison with the reference drug erlotinib having a binding score equal to −9 Kcal/mol. Moreover, the molecular dynamic simulations carried out on compounds 25 and 31 showed that they possessed good stability in the receptor comparable to the standard drug erlotinib. Further preclinical studies should be carried out on the most promising candidates through selecting appropriate *in vitro* and *in vivo* models to validate the efficacy of the compounds. Moreover, convenient drug delivery systems should be developed to ensure the good bioavailability of the compounds as many compounds exhibit good efficacy in preclinical studies, However, they fail in clinical trials owing to their low bioavailability. In addition, suitable analogues could be developed to increase the effectiveness of the compounds. Promising candidates should be subjected to standardization to fall within the criteria of the international standard to develop safe and effective chemopreventive and anticancer products to put an end to suffering from this fatal disease.

## Data Availability

The original contributions presented in the study are included in the article/Supplementary Material, further inquiries can be directed to the corresponding authors.

## Glossary

µg/mL: Microgram per milliliter
µM: Micromole
A375.S2: Malignant melanoma
A549: Adenocarcinomic human alveolar basal epithelial cells
ADME: Absorption, Distribution, Metabolism, Excretion
Ag NPS: Silver nanoparticles
AGS: Gastric cancer
AMJ13: Iraqi breast cancer cell line
Annexin V-FITC: Annexin V-fluorescein isothiocyanate
Au NPS: Gold nanoparticles
Bax: Bcl-2-associated X protein
BBB: Blood brain barrier
Bcl-2: B-cell lymphoma 2
BHK: Baby Hamster kidney cells
**CeO**_**2**_: Cerium oxide nanoparticles
COLO-205: Colon adenocarcinoma cell line
Calu-6: Human lung adenocarcinoma cell line
COX-2: Cyclooxygenase-2
CTNNB1: Catenin Beta 1
CYP 3A4: Cytochrome P450 3A4
DL: Dalton’s lymphoma
DMBA: Dimethylbenz[a]-anthracene
DNA: Deoxyribonucleic acid
EGFR: Epidermal growth factor receptor
EZH2: Enhancer of Zeste 2 Polycomb Repressive Complex 2 Subunit
GI: Gastrointestinal
GSH: Glutathione
GSK-3*β*: Glycogen synthase kinase-3 beta
HBA: Hydrogen bond acceptors
HBD: Hydrogen bond donors
HEK293T: Human embryonic kidney 293T
HCT-116: Human colorectal carcinoma cell line
HeLa 229: Human cervical adenocarcinoma cells
HepG2: Hepatocellular carcinoma
HFB4: Normal human melanocyte cell line
HFF: Human foreskin fibroblast
HT-29: Human colorectal adenocarcinoma cell line
**IC**_**50**_: Half-maximal inhibitory concentration
IL: Interleukin
Jurkat: Human acute myeloid leukemia
K562: Human chronic myeloid leukemia
Kcal/mol: kilocalorie per mole
**LD**_**50**_: Lethal dose, 50%
LNcaP: Prostatic carcinoma cells
Log P: Logarithm of the Partition Coefficient
LRP5: Low-density lipoprotein receptor-related protein 5
LRP6: Low-density lipoprotein receptor-related protein 6
MCF-7: Breast cancer cell line
MDA-MB-231: Breast cancer cell line
MMP-7: Matrix metalloproteinase 7
MNU: N-methyl-N-nitrosourea
MRP2: Multidrug resistance-associated protein
**M**_**W**_: Molecular weight
NCI-H23: Lung cancer cell line
NHDF: Normal human dermal fibroblasts
PC3: Prostate cancer cell line
PHGDH: Phosphoglycerate dehydrogenase
PI: Propidium iodide
ROS: Reactive oxygen species
SK-MEL28: Human melanoma cells
SUIT-2: Human pancreatic carcinoma
TNF: Tumor necrosis factor
TPSA: Topological polar surface area
U87: Human glioblastoma cell
XIAP: X linked inhibitor of apoptosis protein
Y79: Retinoblastoma cell line
