# Supplementary material for: Family Malvaceae: a potential source of secondary metabolites with chemopreventive and anticancer activities supported with in silico pharmacokinetic and pharmacodynamic profiles
Source: Front Pharmacol. 2024 Oct 16;15:1465055. doi: 10.3389/fphar.2024.1465055 (PMC11521888; doi:10.3389/fphar.2024.1465055)
Supplement: Supplementary file 1 [file Table1.DOCX]

***Supplementary Material***

**TABLE S1 Plants’ extracts and fractions prepared from members of family Malvaceae possessing potent anticancer activity**

| **Plant name** | **Common name** | **Geographical distribution** | **Part used** | **Type of extract / Fraction** | **Bioactive phytochemicals** | **Targeted cancer (cell lines)** | **References** |
| --- | --- | --- | --- | --- | --- | --- | --- |
| Abelmoschus esculentus L. | **Lady’s fingers, Red okra** | Tropics of the eastern hemisphere, tropics & subtropics of the western hemisphere | Pulps | Au NPS prepared from aqueous extract | NA | Jurkat | [Mollick et al. (2014](#_ENREF_90)), [Petruzzello (2022](#_ENREF_107)) |
|  |  |  | Flowers | Ag NPS prepared from aqueous extract | NA | A549 & TERT-4 | [Devanesan and AlSalhi, (2021](#_ENREF_47)) |
|  |  |  | Fruits | EtOH extract | Flavonoids, Flavanones & anthocyanins | Breast cancer | [Abdel-Razek et al. (2023](#_ENREF_5)), [Pramudya et al. (2022](#_ENREF_110)) |
|  |  |  | Seeds | NA | Lectin | U87 glioblastoma | [Musthafa et al. (2021](#_ENREF_96)) |
| *Abutilon hirtum* Lam. | Belabenda, Indian mallow | Tropical & south Africa | Leaves | Petroleum ether extract | NA | Ehrlich ascites  carcinoma | [HA ( 2001](#_ENREF_58)) |
| *Adansonia digitata* L. | Baobab | Tropical Africa, southern Arabia, Atlantic & Indian ocean islands | Leaves | Petroleum ether soluble fraction | NA | Brine shrimp lethality assay | Tahia et al. (2015) |
|  |  |  | Bark | MeOH extract |  |  |  |
|  |  |  | Seeds | Aqueous ethanol extract | Glycosides, Saponins, Steroids, Flavonoids | MCF-7, HFB4, BHK | [Abd Alaziz and Abdelmageed (2019](#_ENREF_4)) |
| *Althaea rosea* (L.) Cav. | Hollyhocks | Southern Europe, middle East & central Asia | Flowers | MeOH extract | *trans*-Tiliroside | HepG2 | [Abdel-Salam et al. (2018](#_ENREF_6)) |

**NA**: not available, **NPS**: nanoparticles

**TABLE S1 Plants’ extracts and fractions prepared from members of family Malvaceae possessing potent anticancer activity (Cont.)**

| **Plant name** | **Common name** | **Geographical distribution** | **Part used** | **Type of extract / Fraction** | **Bioactive phytochemicals** | **Targeted cancer (cell lines)** | **References** |
| --- | --- | --- | --- | --- | --- | --- | --- |
| *Brachychiton discolor* F. Muell. | Lace kurrajong, Pink kurrajong, Scrub bottle tree, White kurrajong | Australia | Flowers | Essential oils | Palmitic acid | MCF-7, A549 | [Thabet et al. (2020](#_ENREF_148)) |
| *Brachychiton rupestris* (T.Mitch. ex Lindl.) K. Schum. | Queensland bottle tree | Australia | Leaves | Essential oils | *δ*−Cadinene & *β*-Caryophyllene | HepG2 | [Thabet et al. (2020](#_ENREF_148)) |
| *Cola gigantea* A. Chev. | Giant cola | Tropical west Africa | Seeds | Essential oils | Campesterol, Stigmasterol & *β*-Sitosterol | HFF | [Atolani et al. (2019](#_ENREF_27)) |
| *Corchorus olitorius* L. | Jute mallow, Nalta jute | Tropical & subtropical regions | Aerial parts | Aqueous EtOH extract | Phenolics & Flavonoids | CaCi 1962, LiGh 1927B & SK-MEL28 | [Handoussa et al. (2013](#_ENREF_59)) |
|  |  |  | Leaves | Gold NPS & iron oxide NPS prepared from 70% EtOH extract | Phenolics & Flavonoids | MCF-7 | [El-Rafie et al. (2016](#_ENREF_49)) |
|  |  |  | Whole plant | MeOH extract | Phenolics & Flavonoids | MCF-7 & A549 | [Alshabi et al. (2022](#_ENREF_19)) |
| *Hibiscus cannabinus* L. | Kenaf, Java jute | Africa, South Asia | Bark | Acetone extract | Boehmenan H & *threo*-Carolignan K | HeLa, Hep-2 & A549 | [Moujir et al. (2007](#_ENREF_93)) |

**NPS**: nanoparticles

**TABLE S1 Plants’ extracts and fractions prepared from members of family Malvaceae possessing potent anticancer activity (Cont.)**

| **Plant name** | **Common name** | **Geographical distribution** | **Part used** | **Type of extract / Fraction** | **Bioactive phytochemicals** | **Targeted cancer (cell lines)** | **References** |
| --- | --- | --- | --- | --- | --- | --- | --- |
| *Hibiscus syriacus* L. | Rose of Sharon, Shrub althea | South-central & southeast China, north America | Root bark | Acetone extract | Betulin 3-caffeate | A549 | [Shi et al. (2014](#_ENREF_130)) |
|  |  |  | Leaves | 70% EtOH callus extract | NA | HT-29 xenograft | [Xu et al. (2022](#_ENREF_156)) |
| *Hibiscus tiliaceus* L. | Sea Hibiscus, Beach Hibiscus, Linden Hibiscus, Cotton Tree, BaruBaru, Bebaru, Mahoe | Tropics of Africa, America, Asia, Australia & Pacific islands | Stem wood | MeOH extract | Hibiscusamide & *N*-*trans*-Feruloyltyramine | P-388 & HT-29 | [Chen et al. (2006](#_ENREF_37)) |
|  |  |  | Leaves | EtOAC extract | NA | MCF-7 | [Andriani et al. (2020](#_ENREF_24)) |
| *Sida acuta* Burm. F. | Wireweed, Teaweed, Ironweed | Central America, tropics & sub-tropics in the Pacific, Asia, Africa | Whole plant | EtOAC extract | Cryptolepinone & *N*-*trans*-Feruloyltyramine | Mouse mammary organ culture | [Jang et al. (2003](#_ENREF_72)) |
|  |  |  | Whole plant | MeOH extract | Cryptolepine | AGS | [Ahmed et al. (2011](#_ENREF_12)) |

**NA**: not available

**TABLE S2 Plants’ extracts and fractions prepared from members of family Malvaceae possessing moderate or weak anticancer activity**

| **Plant name** | **Common name** | **Geographical distribution** | **Part used** | **Type of extract / Fraction** | **Bioactive phytochemicals** | **Targeted cancer (cell lines)** | **References** |
| --- | --- | --- | --- | --- | --- | --- | --- |
| Abelmoschus esculentus L. | **Lady’s fingers, Red okra** | Tropics of the eastern hemisphere,tropics & subtropics of the western hemisphere | Flowers | EtOAC fraction | NA | HepG2 | Solomon et al. (2016) |
|  |  |  | Fruits | Polysaccharide extract | Polysaccharides | Huh7it | Hayaza et al. (2019) |
|  |  |  |  | CeO_2_ NPS | NA | HeLa | Ahmed et al. (2021) |
| *Abelmoschus moschatus* Medik. | **Musk mallow, Abelmosk,**  **Ambrette, Muskdana, Musk okra** | Southeast Asia, northern Australia, tropical Asia, Madagascar, central and south America, the Caribbean, Europe & many islands in the Pacific region | Aerial parts | EtOH & Acetone extracts | Phytol, Loliolide & *ᵧ*- Sitosterol | HepG2 | Sebastian et al. (2022) |
|  |  |  | Leaves & Seeds | Hydroalcohol extract | Polyphenolics & Flavonoids | Colorectal adenocarcinoma (COLO-205) & retinoblastoma (Y79) | Gul et al. (2011), Rojas-Sandoval, (2018) |
| *Abutilon grandiflorum* G. Don | Hairy Indian mallow | Tropical America, central & south Africa | Roots | EtOAC extract | NA | HT-29 | Beha et al. (2004) |
|  |  |  |  | Petroleum ether extract |  |  |  |
|  |  |  |  | Aqueous extract |  |  |  |
| *Abutilon hirtum* Lam. | Belabenda, Indian mallow | Tropical & south Africa | Flowers | Aqueous extract | Phenols & Flavonoids | MCF-7 | Wesley et al. (2013) |
| *Abutilon indicum* L. | India Abutilon, Monkey Bush | Tropical & subtropical zones | Leaves | EtOAC fraction | Methyl caffeate | U87MG human glioblastoma | Khan et al. (2015) |
|  |  |  | Whole plant | Methanol extract | Phenols and Flavonoids | SK-MEL28 & NCI-H23 | Srikanth et al. (2012) |

**NA**: not available, **NPS**: nanoparticles

**TABLE S2 Plants’ extracts and fractions prepared from members of family Malvaceae possessing moderate or weak anticancer activity (Cont.)**

| **Plant name** | **Common name** | **Geographical distribution** | **Part used** | **Type of extract / Fraction** | **Bioactive phytochemicals** | **Targeted cancer (cell lines)** | **References** |
| --- | --- | --- | --- | --- | --- | --- | --- |
| *Adansonia digitata* L. | Baobab | Tropical Africa, southern Arabia, Atlantic & Indian ocean islands | Fruits | Ag NPS & aqueous extract | NA | HTC116 & SW480 | Almukaynizi et al. (2022) |
| Althaea officinalis L. | Marsh mallow | Europe, western Asia & north Africa | NA | Crude extract | Flavonoids, & Phytosterols | AMJ 13 | Kadhum et al. (2021) |
|  |  |  | Flowers | Aqueous extract | Polysaccharides, Flavonoids, Phenolic Acids & Coumarins | A549, EB, HCT116 p53 null cells, p53 wild type HCT116, MCF-7, HeLa 229 and NHDF | Farhat et al. (2022) |
| *Brachychiton populneus* (Schott & Endl.) R.Br. | Kurrajong, Bottelboom | Australia | Leaves | Ag NPS prepared from aqueous extract | NA | U87 glioblastoma & HEK 293 | Naveed et al. (2022) |
| *Corchorus olitorius* L. | Jute mallow, Nalta jute | Tropical & subtropical regions | Whole plant | MeOH extract | Methyl-1,4,5-*tri*-*O*-caffeoyl quinate | HeLa | Taiwo et al. (2016) |
| *Hibiscus ficulneus* L. | White wild musk mallow, Native rosella | North and east Africa, Madagascar, Indomalaya, north Australia | Stem | MeOH extract | Boehmenan | Wnt-independent (RKO) & Wnt-dependent (HCT116) | Shono et al. (2015) |

**NA**: not available, **NPS**: nanoparticles

**TABLE S2 Plants’ extracts and fractions prepared from members of family Malvaceae possessing moderate or weak anticancer activity (Cont.)**

| **Plant name** | **Common name** | **Geographical distribution** | **Part used** | **Type of extract / Fraction** | **Bioactive phytochemicals** | **Targeted cancer (cell lines)** | **References** |
| --- | --- | --- | --- | --- | --- | --- | --- |
| *Hibiscus sabdariffa* L. | Roselle, Jamaica Sorrel, Red Sorrel, Sorrel, Indian Sorrel, Asam Susar | Angola, Egypt, Guatemala, Guinea, India, Myanmar, Nigeria, Philippines Jamaica, Mexico, Spain, United States of America | Calyx | EtOH extract | NA | MCF-7 & MDA-MB231 | Malacrida et al. (2022) |
| *Hibiscus syriacus* L. | Rose of Sharon, Shrub althea | South-central & southeast China, north America | Root bark | NA | Betulin & Betulinic acid | MDA-MB231 & HBL-100 | Hsu et al. (2015) |
| *Hibiscus tiliaceus* L. | Sea Hibiscus, Beach Hibiscus, Linden Hibiscus, Cotton Tree, BaruBaru, Bebaru, Mahoe | Tropics of Africa, America, Asia, Australia & Pacific islands | Stems & twigs | MeOH extract | Syriacusin A & Hibiscone C | HeLa | Matsumoto et al. (2020) |
| *Malva parviflora* L. | Egyptian mallow, Little mallow, Cheeseweed | North Africa, Europe, Asia | Stems | Hexane extract | NA | MDA-MB231 | Singh (2017), Nasr et al. (2018) |
| *Malva sylvestris* L. | Common mallow, Wood mallow, Tree mallow, High mallow | Europe, western Asia, northern Africa | NA | Fe_3_O_4_ NPS | NA | MCF-7 & HepG2 | Mousavi et al. (2020) |

**NA**: not available, **NPS**: nanoparticles

**TABLE S2 Plants’ extracts and fractions prepared from members of family Malvaceae possessing moderate or weak anticancer activity (Cont.)**

| **Plant name** | **Common name** | **Geographical distribution** | **Part used** | **Type of extract / Fraction** | **Bioactive phytochemicals** | **Targeted cancer (cell lines)** | **References** |
| --- | --- | --- | --- | --- | --- | --- | --- |
| *Pavonia odorata* Willd. | Fragrant swamp mallow | India, Pakistan, Burma, Srilanka, East tropical Africa. | Whole plant | MeOH extract | Flavonoids | MD-MB231, PC3 & Calu-6 | Girish et al. (2016) |
| *Pavonia xanthogloea* Ekman | Erva-de-ovelha | Brazil | Aerial parts | Aqueous fraction | Phenolic compounds | Human lymphocytes | Mostardeiro et al. (2014) |
| *Wissadula periplocifolia* (L.) C. Presl | White velvet leaf | Tropical areas of America & Asia | Aerial parts | EtOH extract | Acacetin, Tiliroside,  7-*O*-sulphate acacetin (wissadulin) + 4′-*O*-methyl-8-*O*-sulphate isoscutellarein (caicoine) | UVW glioma & PC-3M | Teles et al. (2015) |

**TABLE S3 Bioactive metabolites in family Malvaceae and their anticancer mechanisms of action**

| **Compound name** | **Mechanism of action** | **Activity** | **Standard drug** | **Plant name** | **Part used** | **References** |
| --- | --- | --- | --- | --- | --- | --- |
| **Carotenoids** | | | | | | |
| *β*-Carotene (**1**) | Activates caspase-3, decreases Bcl2, PARP & NF-κB, inhibits (ERK1/2 & Akt), apoptosis | -Active at a concentration of 1 µM against MCF-7 (in vitro) | NA | Abelmoschus esculentus | Fruits | [Abdel-Razek et al. (2023](#_ENREF_5)), [Sowmya et al. (2017](#_ENREF_139)) |
|  |  |  |  | *Hibiscus syriacus* | Seeds | [Azimova et al. (2011](#_ENREF_2)), [Sowmya et al. (2017](#_ENREF_139)) |
| Lycopene (**2**) | Inhibits phosphorylation of Akt, GSK-3*β* & ERK 1/2, inhibits (*c-myc*, cyclin E, Ras, NF-κB, phosphorylation of c-jun N-terminal kinase, extracellular signal-regulated kinase 1/2 & p38), decreases cyclin D1, increases (p21, p27, p53 & Bax:Bcl-2 ratio), anti-metastasis, antiangiogenic | -Active at a concentration of 2 µM against HT-29 (in vitro)  -Active at concentrations (2.5-10 µM) against LNCaP, PC-3, HCT-116 and HT-29 (in vitro) | NA | Abelmoschus esculentus | Seeds | [Abdel-Razek et al. (2023](#_ENREF_5)), [Lin et al. (2011](#_ENREF_83)), [Park et al. (2019](#_ENREF_105)) [Palozza et al. (2010](#_ENREF_104)) |
| Zeaxanthin (**3**) | Apoptosis, cell cycle arrest at G2/M phase | -IC_50_ = 17 µM against AGS (in vitro) | 5- Fluorouracil  -IC_50_ = 23.34 µM against AGS (in vitro) | Abelmoschus esculentus | Fruits | [Abdel-Razek et al. (2023](#_ENREF_5)), [Sheng et al. (2020](#_ENREF_129)) |

**NA**: not available

**TABLE S3 Bioactive metabolites in family Malvaceae and their anticancer mechanisms of action (Cont.)**

| **Compound name** | **Mechanism of action** | **Activity** | **Standard drug** | **Plant name** | **Part used** | **References** |
| --- | --- | --- | --- | --- | --- | --- |
| **Phenolic acid derivatives** | | | | | | |
| Caffeic acid (**4**) | Alters p53, p21 & Mcl-1 genes expression, inhibits CDK2, CDK3 & CDK4, arrests cell cycle at G1 or G2 phases, decreases MMP-2 & MMP-9 expression, decreases HIF-1*α* activity, apoptosis | -IC_50_ = 159 and 3 µg/ml against MCF-7 and Huh-7, respectively (in vitro) | Tamoxifen citrate  -IC_50_ = 16 µg/ml against MCF-7 (in vitro)  Doxorubicin  -IC_50_ = 2 µM against Huh-7 (in vitro) | Abelmoschus esculentus | Leaves | [Abdel-Razek et al. (2023](#_ENREF_5)), [Rezaei-Seresht et al. (2019](#_ENREF_115)), [El-Din et al. (2014](#_ENREF_48)) |
|  |  |  |  | *Abutilon indicum* | Whole plant, leaves & aerial parts | [Gomaa et al. (2018](#_ENREF_56)) [Rezaei-Seresht et al. (2019](#_ENREF_115)), [El-Din et al. (2014](#_ENREF_48)) |
|  |  |  |  | *Althaea officinalis* | Flower | [Farhat et al. (2022](#_ENREF_50)), [Rezaei-Seresht et al. (2019](#_ENREF_115)), [El-Din et al. (2014](#_ENREF_48)) |
|  |  |  |  | *Corchorus olitorius* | Leaves | [Oboh et al. (2012](#_ENREF_100)), [Rezaei-Seresht et al. (2019](#_ENREF_115)), [El-Din et al. (2014](#_ENREF_48)) |
|  |  |  |  | *Pavonia xanthogloea* | Aerial parts | [Mostardeiro et al. (2014](#_ENREF_92)), [Rezaei-Seresht et al. (2019](#_ENREF_115)), [El-Din et al. (2014](#_ENREF_48)) |

**TABLE S3 Bioactive metabolites in family Malvaceae and their anticancer mechanisms of action (Cont.)**

| **Compound name** | **Mechanism of action** | **Activity** | **Standard drug** | **Plant name** | **Part used** | **References** |
| --- | --- | --- | --- | --- | --- | --- |
| **Phenolic acid derivatives (Cont.)** | | | | | | |
| Chlorogenic acid (**5**) | Cell cycle arrest at S phase, decreases phosphorylation of ERK1/2, decreases MMP-2 and MMP-9 expression, decreases Bcl-2, increases Bax, activates caspase-3 | -IC_50_ = 306.64, 330.74 and 373.53 µM against A-375, AGS and SUIT-2, respectively (in vitro)  -Active at concentrations (30 and 50 µM) against A549 (in vitro)  -Active at doses 30 and 60 mg/kg b.w.in vivo against HepG2 xenografts | NA | Abelmoschus esculentus | Leaves | [Abdel-Razek et al. (2023](#_ENREF_5)), [Yan et al. (2017](#_ENREF_160)), [Yamagata et al. (2018](#_ENREF_158)) |
|  |  |  |  | *Corchorus olitorius* | Leaves | [Tosoc et al. (2021](#_ENREF_150)), [Yan et al. (2017](#_ENREF_160)), [Yamagata et al. (2018](#_ENREF_158)) |
|  |  |  |  | *Pavonia xanthogloea* | Aerial parts | [Mostardeiro et al. (2014](#_ENREF_92)), [Yan et al. (2017](#_ENREF_160)), [Yamagata et al. (2018](#_ENREF_158)) |
| *p*-Coumaric acid (**6**) | Cell cycle arrest at sub-G1 phase, apoptosis, antiangiogenic, down-regulates VEGF, AKT and ERK signalling pathways | -IC_50_ = 1400, 1600 µM/l against HCT-15 and HT-29 (in vitro)  -Active at a concentration of 5 mM against ECV304 (in vitro)  -Active at dose of 150 mg/kg b.w. in vivo in mouse tumor model injected with RENCA cells | NA | Abelmoschus esculentus | Leaves | [Abdel-Razek et al. (2023](#_ENREF_5)), [Jaganathan et al. (2013](#_ENREF_71)), [Kong et al. (2013](#_ENREF_77)) |
|  |  |  |  | *Abutilon hirtum* | Non flowering aerial parts | [Gomaa et al. (2018](#_ENREF_56)) , [Jaganathan et al. (2013](#_ENREF_71)), [Kong et al. (2013](#_ENREF_77)) |
|  |  |  |  | *Abutilon indicum* | Whole plant &aerial parts | [Gomaa et al. (2018](#_ENREF_56)) , [Jaganathan et al. (2013](#_ENREF_71)), [Kong et al. (2013](#_ENREF_77)) |

**NA**: not available

**TABLE S3 Bioactive metabolites in family Malvaceae and their anticancer mechanisms of action (Cont.)**

| **Compound name** | **Mechanism of action** | **Activity** | **Standard drug** | **Plant name** | **Part used** | **References** |
| --- | --- | --- | --- | --- | --- | --- |
| **Phenolic acid derivatives (Cont.)** | | | | | | |
| Eugenol (**7**) | Scavenges nitric oxide radicals | NA | NA | Abelmoschus esculentus | Pods | [Abdel-Razek et al. (2023](#_ENREF_5)), [Sharma et al. (2017](#_ENREF_128)) |
| *N*-*trans*-Feruloyltyramine (**8**) | Inhibits the oxidative damage caused by H_2_O_2_, conserves the integrity of mitochondrial membrane | - IC_50_ = 2.3 and 5.7 µg/ml against P-388 and HT-29, respectively (in vitro)  -IC_50_ = 194 µM against HepG2(in vitro) | Mithramycin  - IC_50_ = 0.06 and 0.08 µg/ml against P-388 and HT-29, respectively (in vitro) | *Hibiscus tiliaceus* | Stem wood | [Chen et al. (2006](#_ENREF_37)), [Gao et al. (2019](#_ENREF_53)) |
|  |  |  |  | *Sida acuta* | Whole plant | [Jang et al. (2003](#_ENREF_72)), [Gao et al. (2019](#_ENREF_53)) |
| Gallic acid (**9**) | Increases tumor suppressor gene p53 & Bax, decreases Bcl-2 & GSH, activates caspase-3 & -9, increases ROS, apoptosis, alters p53, p21 and Mcl-1 genes expression | -IC_50_ = 18 µg/ml against MCF-7 (in vitro)  -IC_50_ = 50 µM/l and 80 µM against SMMC-7721 and HeLa, respectively (in vitro)  -Active at a concentration of 250 µM against A375.S2 (in vitro) | Tamoxifen citrate  -IC_50_ = 16 µg/ml against MCF-7 (in vitro) | Abelmoschus esculentus | Roots | [Abdel-Razek et al. (2023](#_ENREF_5)), [Rezaei-Seresht et al. (2019](#_ENREF_115)), [Subramanian et al. (2015](#_ENREF_141)), [Lo et al. (2010](#_ENREF_86)), [You et al. (2010](#_ENREF_163)) |
|  |  |  |  | *Abutilon indicum* | Whole plant, leaves and aerial parts | [Gomaa et al. (2018](#_ENREF_56)) , [Rezaei-Seresht et al. (2019](#_ENREF_115)), [Subramanian et al. (2015](#_ENREF_141)), [Lo et al. (2010](#_ENREF_86)), [You et al. (2010](#_ENREF_163)) |
|  |  |  |  | *Althaea officinalis* | Flowers | [Farhat et al. (2022](#_ENREF_50)), [Rezaei-Seresht et al. (2019](#_ENREF_115)), [Subramanian et al. (2015](#_ENREF_141)), [Lo et al. (2010](#_ENREF_86)), [You et al. (2010](#_ENREF_163)) |

**TABLE S3 Bioactive metabolites in family Malvaceae and their anticancer mechanisms of action (Cont.)**

| **Compound name** | **Mechanism of action** | **Activity** | **Standard drug** | **Plant name** | **Part used** | **References** |
| --- | --- | --- | --- | --- | --- | --- |
| **Phenolic acid derivatives (Cont.)** | | | | | | |
| Hibiscusamide (**10**) | Reduces p-STAT3, p-JAK2 & p-ERK phosphorylation, inhibits IL-6 signalling pathway | *-*IC_50_ = 1.7 and 3.8 µg/ml against P-388 and HT-29, respectively (in vitro)  -IC_50_ = 0.2 µM against Hep3B (in vitro) | Mithramycin  - IC_50_ = 0.06 and 0.08 µg/ml against P-388 and HT-29, respectively (in vitro) | *Hibiscus tiliaceus* | Stem wood | [Chen et al. (2006](#_ENREF_37)), [Hwang et al. (2016](#_ENREF_70)) |
| Methyl caffeate (**11**) | Inhibits PHGDH enzyme, apoptosis | -IC_50_ = 8.2 µg/ml against U87MG (in vitro) | NA | *Abutilon indicum* | Leaves | [Khan et al. 2015](#_ENREF_75), [Gomaa et al. (2018](#_ENREF_56)) , [Wang et al. (2021](#_ENREF_153)) |
| Methyl-1,4,5-tri-*O*-caffeoyl quinate (**12**) | NA | -Active at a concentration of 800 µM against HeLa (in vitro) | NA | *Corchorus olitorius* | Whole plant | [Taiwo et al. (2016](#_ENREF_144)) |
| Rosmarinic acid (**13**) | At low concentration: (antioxidant effect, reduces cell proliferation), at high concentration: (prooxidant activity, necrosis, cell death), inhibits MARK4 & proteasome, apoptosis | -LC_50_ = 290.5 µM against glioblastoma (in vitro)  -Inhibits MARK4 activity with IC_50_ = 6.2 µM (in vitro)  -Active at a concentration of 1000 µM against HepG2 (in vitro) | NA | Abelmoschus esculentus | Leaves | [Abdel-Razek et al. (2023](#_ENREF_5)), [Ramanauskiene et al. (2016](#_ENREF_114)), [Anwar et al. (2020](#_ENREF_26)), [Ozgun and Ozgun, (2020](#_ENREF_103)) |
| Syriacusin A (**14**) | Reduces cell proliferation, apoptosis | -IC_50_ = 42.3 µM against HeLa (in vitro) | Adriamycin  -IC_50_ = 1.48 µM against HeLa (in vitro) | *Hibiscus tiliaceus* | Stems & twigs | [Μatsumoto et al. (2020](#_ENREF_165)) |
| Syringic acid (**15**) | Increases caspases-3 & -9, cytochrome-c, Apaf-1, Bax & p53, decreases Bcl-2, apoptosis | -IC_50_ = 40.54 µM against HepG2 (in vitro) | NA | *Abutilon indicum* | Leaves | [Khan et al. 2015](#_ENREF_75), [Gheena and Ezhilarasan, (2019](#_ENREF_54)) |

**NA**: not available

**TABLE S3 Bioactive metabolites in family Malvaceae and their anticancer mechanisms of action (Cont.)**

| **Compound name** | **Mechanism of action** | **Activity** | **Standard drug** | **Plant name** | **Part used** | **References** |
| --- | --- | --- | --- | --- | --- | --- |
| **Flavonoids** | | | | | | |
| Acacetin (**16**) | Reduces topoisomerase 1 & tyrosinase activity, increases p53, cell cycle arrest at G1 & or G2/M phase, activates caspase-7, apoptosis, inhibits MMP-2 and -9 | -IC_50_ = 21.13 and 27.37 µg/ml against PC-3M and UVW glioma, respectively (in vitro)  - Active at dose of 5 mg/kg b.w.in vivo in SK-MeL-28 cell induced tumor model  - Active at a dose of 50 mg/kg b.w. in vivo in chronic lymphocytic leukemia | NA | *Wissadula periplocifolia* | Aerial parts | [Teles et al. (2015](#_ENREF_145)), [Singh et al. (2020](#_ENREF_136)) |
| Apigenin (**17**) | Arrests cell cycle at G2/M phase, decreases cyclin B1, Cdc2 & Cdc25c expression, up-regulates p53 & p21 expression, down-regulates Bcl-2, Bcl-xL, Bcl-w & Mcl-1, up-regulates Bad, Bak, Bax, Bid, & Bim, decreasesMMP-9, reduces myeloperoxidase, decreases inflammatory cytokine & COX-2, inhibits NF-κB & STAT3, decreases inflammation | -IC_50_ = 35.15, 10, 68, 76 and 40 µM against MDA-MB-453, HeLa, SiHa, CaSki and C33A, respectively (in vitro) | NA | Abelmoschus esculentus | Pods | [Pramudya et al. (2022](#_ENREF_110)), [Choi and Kim (2009](#_ENREF_41)), [Souza et al. (2017](#_ENREF_138)), [Ai et al., (2017](#_ENREF_14)), [Ashrafizadeh et al. (2020](#_ENREF_4)) |
|  |  |  |  | *Althaea officinalis* | Flowers | [Farhat et al. (2022](#_ENREF_50)), [Choi and Kim (2009](#_ENREF_41)), [Souza et al. (2017](#_ENREF_138)), [Ai et al., (2017](#_ENREF_14)) |
|  |  |  |  | *Althaea rosea* | Flowers | [Abd El-Salam et al. (2016](#_ENREF_3)), [Choi and Kim (2009](#_ENREF_41)), [Souza et al. (2017](#_ENREF_138)), [Ai et al., (2017](#_ENREF_14)) |

**NA**: not available

**TABLE S3 Bioactive metabolites in family Malvaceae and their anticancer mechanisms of action (Cont.)**

| **Compound name** | **Mechanism of action** | **Activity** | **Standard drug** | **Plant name** | **Part used** | **References** |
| --- | --- | --- | --- | --- | --- | --- |
| **Flavonoids (Cont.)** | | | | | | |
| Astragalin (**18**) | Down-regulates MMP-2 & MMP-9 expression, increases caspase-3, -6, -7, -8, -9, & P53, inhibits CDK2, CDK4, cyclin D1 and cyclin E, elevates Bax:Bcl-2 ratio, reduces extracellular signal-regulated kinase (ERK)-1/2 &Akt signalling, reduces (TNFα)-induced NF-κB activity, apoptosis | -IC_50_ = 18.88 µg/ml against HCT 116 (in vitro)  -Active at a concentration of 40 µg/ml against A549 (in vitro)  - Active at dose of 50 mg/kg b.w. in vivo in A549 xenograft tumor model | NA | *Corchorus olitorius* | Leaves | [Hasan and Kadhim (2018](#_ENREF_61)), [Yang et al. (2021](#_ENREF_27))  [Chen et al. (2017](#_ENREF_38)) |
| Caicoine (**19**) | NA | NA | NA | *Wissadula periplocifolia* | Aerial parts | [Teles et al., (2015](#_ENREF_145)). [Teles et al. (2018](#_ENREF_146)) |
| Chrysin (**20**) | Apoptosis, reduces NF-κB activation, down-regulates anti-apoptotic NF-κB target gene, induces caspase-3, down-regulates XIAP | -IC_50_ = 19.5 µM against MCF-7(in vitro)  -IC_50_ = 18.51 µg/ml against Huh-7 (in vitro) | Doxorubicin  -IC_50_ = 2 µM against Huh-7 (in vitro) | *Althaea officinalis* | Flowers | [Farhat et al. (2022](#_ENREF_50)), [El-Din et al. (2014](#_ENREF_48)), [Samarghandian et al. (2011](#_ENREF_29)), [Samarghandian et al. (2016](#_ENREF_122)) |
| Galangin (**21**) | Increases p53 & caspase-8, apoptosis | -IC_50_ = 88.4 µg/ml against SKOV3 (in vitro) | NA | *Althaea officinalis* | Flowers | [Farhat et al. (2022](#_ENREF_50)), ([Al-Shammari et al. (2020](#_ENREF_16)) |
| Isoquercetin (**22**) | Reduces phosphorylation of protein kinase B, enhances activity of caspases, decreases BCl-2 & MCl-1, scavenges ROS | -IC_50_ = 369.29, 355.97 and 144.45 µM against A-375, AGS and SUIT-2, respectively (in vitro) | NA | *Corchorus olitorius* | Leaves | [Tosoc et al. (2021](#_ENREF_150)), [Akter et al. (2021](#_ENREF_15)) |
| Kaempferol (**23**) | Decreases cyclin D1, cyclin E, cathepsin D, pIRS-1, pAkt & pMEK1/2 expression, up-regulates p21 & Bax | -Active at a concentration of 50 µM against MCF-7 (in vitro)  -Active at a dose of 100 mg/kg b.w. in MCF-7 transplanted mouse model | NA | *Althaea officinalis* | Flowers | [Farhat et al. (2022](#_ENREF_50)), [Kim et al. (2016](#_ENREF_76)) |

**NA**: not available

**TABLE S3 Bioactive metabolites in family Malvaceae and their anticancer mechanisms of action (Cont.)**

| **Compound name** | **Mechanism of action** | **Activity** | **Standard drug** | **Plant name** | **Part used** | **References** |
| --- | --- | --- | --- | --- | --- | --- |
| **Flavonoids (Cont.)** | | | | | | |
| Quercetin (**24**) | Decreases MMP9, MMP2, p-EGFR, VEGFR-2, p-PI3K, Akt & pGSK3ß expression, stops NF-κB activity, inhibits MAPK pathway | -IC_50_ = 48.61 µg/ml against U251 (in vitro) | NA | Abelmoschus esculentus | Pods | [Pramudya et al. (2022](#_ENREF_110)), [Liu et al. (2017](#_ENREF_85)) |
|  |  |  |  | *Althaea officinalis* | Flowers | [Farhat et al. (2022](#_ENREF_50)), [Liu et al. (2017](#_ENREF_85)) |
|  |  |  |  | *Corchorus olitorius* | Leaves | [Yakoub et al. (2018](#_ENREF_157)), [Liu et al. (2017](#_ENREF_85)) |
| Tiliroside (**25**) | Inhibits CAXII, decreases E2F1 & E2F3 expression, activates caspases-3, -8 & -9, decreases the expression of Bcl-2 protein | -IC_50_ = 3.822, 60.55, 67.79 and 100 µg/ml against HepG2, PC-3M, T47D and MCF-7, respectively (in vitro) | 5- Fluorouracil  -IC_50_ = 0.9 µg/ml against HepG2 (in vitro) | *Althaea rosea* | Flowers | [Abdel-Salam et al. (2018](#_ENREF_6)), [Da'i et al. (2016](#_ENREF_44)) |
|  |  |  |  | *Wissadula periplocifolia* | Aerial parts | [Teles et al., (2015](#_ENREF_145)), [Da'i et al. (2016](#_ENREF_44)) |
| Wissadulin (**26**) | NA | NA | NA | *Wissadula periplocifolia* | Aerial parts | [Teles et al., (2015](#_ENREF_145)), [Teles et al. (2018](#_ENREF_146)) |
| **Coumarins** | | | | | | |
| Scopoletin (**27**) | Apoptosis, activates caspase-3, cell cycle arrest at G_2_/M phase, blocks PI3K/Akt/mTOR signalling pathway, down-regulates cyclin D1 expression | -IC_50_ = 65.1 µM against LNCaP (in vitro) | NA | *Althaea officinalis* | Leaves | [Kadhum](#_ENREF_73) et al. (2021), [Shah et al., (2011](#_ENREF_127)), [Li et al. (2015](#_ENREF_81)), [Meilawati et al. (2023](#_ENREF_22)) |
| **Alkaloids** | | | | | | |
| Cryptolepine (**28**) | Affects cyclin D1, D2, D3 & cyclin E, controls IGF-IR expression, regulates PI3k/Akt pathway | NA | NA | *Sida acuta* | Whole plant | [Ahmed et al. (2011](#_ENREF_12)), [Ansha and Mensah (2013](#_ENREF_25)) |
| Cryptolepinone (**29**) | Induces quinone reductase | -Active at a dose of 10 µg/ml against Hepa 1c1c7 | NA | *Sida acuta* | Whole plant | [Jang et al. (2003](#_ENREF_72)) |

**NA**: not available

**TABLE S3 Bioactive metabolites in family Malvaceae and their anticancer mechanisms of action (Cont.)**

| **Compound name** | **Mechanism of action** | **Activity** | **Standard drug** | **Plant name** | **Part used** | **References** |
| --- | --- | --- | --- | --- | --- | --- |
| **Lignans** | | | | | | |
| Boehmenan (**30**) | Inhibits cytosolic, nuclear *β*-catenin & *c-myc* expression, reduces Wnt signal | -IC_50_ = 25.4 and 22.8 µM against Wnt-independent (RKO) and Wnt-dependent cells (HCT116), respectively (in vitro) | NA | *Hibiscus ficulneus* | Stems | [Shono et al. (2015](#_ENREF_131)) |
| Boehmenan H (**31**) | NA | -IC_50_ = 7.7, 10.2, 10.7 and 4.2 µg/ml against LNCaP, MCF-7, Hep-2 and A549, respectively (in vitro) | Actinomycin D  -IC_50_ = 0.008 µg/ml against Hep-2 (in vitro) | *Hibiscus cannabinus* | Bark | [Moujir et al. (2007](#_ENREF_93)), [Pham et al. (2021](#_ENREF_20)) |
| *threo*-Carolignan K (**32**) | NA | -IC_50_ = 3.3 and 3.7 µg/ml against Hep-2 and A549, respectively (in vitro) | Actinomycin D  -IC_50_ = 0.008 µg/ml against Hep-2 (in vitro) | *Hibiscus cannabinus* | Bark | [Moujir et al. (2007](#_ENREF_93)) |
| **Cardiac glycosides** | | | | | | |
| Glucoevatromonoside (**33**) | Induces apoptosis, cell cycle arrest at G2/M phases, down-regulates cyclin B1 | -IC_50_ = 19.3 nM against A549 (in vitro) | Paclitaxel  -IC_50_ = 260.5 nM against A549 (in vitro) | *Corchorus olitorius* | Seeds | [Biswas et al. (2022](#_ENREF_5)), [Schneider et al. (2018](#_ENREF_125)) |
| Helveticoside (**34**) | Increases Bax level, inhibits Bcl-2 , cleaves caspase-3 and -9, apoptosis | -Active at concentrations (0.6, 0.8 and 1 mM) against SW480 and HCT116 (in vitro)  -Active at doses (1 and 2 mg/kg b.w.) in colorectal cancer xenograft model | NA | *Corchorus olitorius* | Seeds | [Biswas et al. (2022](#_ENREF_5)), [An et al. (2020](#_ENREF_21)) |
| **Sterols & Terpenes** | | | | | | |
| Betulin (**35**) | Apoptosis, increases p21 level | -Active at concentration (10 µg/ml) against MDA-MB-231 and HBL-100 (in vitro) | NA | *Hibiscus syriacus* | Root bark | [Hsu et al. (2015](#_ENREF_68)) |

**NA**: not available

**TABLE S3 Bioactive metabolites in family Malvaceae and their anticancer mechanisms of action (Cont.)**

| **Compound name** | **Mechanism of action** | **Activity** | **Standard drug** | **Plant name** | **Part used** | **References** |
| --- | --- | --- | --- | --- | --- | --- |
| **Sterols & Terpenes (Cont.)** | | | | | | |
| Betulinic acid (**36**) | Activates mitochondrial apoptotic pathway, increases p21 level, increases ROS, inhibits topoisomerase I, II*α*, inhibits angiogenesis, down-regulates aminopeptidase N | -Active at concentration (10 µg/ml) against MDA-MB-231 and HBL-100 (in vitro)  - IC_50_ = 9.8 and 1.2 µg/ml against MCF-7 and HT-29, respectively (in vitro) | NA | *Hibiscus syriacus* | Root bark | [Hsu et al. (2015](#_ENREF_68)), [Hordyjewska et al. (2019](#_ENREF_65)) |
|  |  |  |  | *Corchorus olitorius* | Leaves | [Ramadevi (2013](#_ENREF_113)), [Hordyjewska et al. (2019](#_ENREF_65)) |
| Betulin-3-caffeate (**37**) | NA | -IC_50_ = 4.3 µM against A549 (in vitro) | Taxol  -IC_50_ = 2.4 nM against A549 (in vitro) | *Hibiscus syriacus* | Root bark | [Shi et al. (2014](#_ENREF_130)) |
| *δ*−Cadinene (**38**) | NA | -IC_50_ = 3.86 and 3.66 µg/ml against BT-20 and HeLa (in vitro) | NA | *Brachychiton rupestris* | Leaves | [Thabet et al. (2020](#_ENREF_148)), [Ali et al. (2017](#_ENREF_17)), [Kubo and Morimitsu (1995](#_ENREF_79)) |
| Campesterol (**39**) | Antiangiogenic | NA | NA | *Althaea officinalis* | NA | [Kadhum et](#_ENREF_73) al. (2021), [Choi et al. (2007](#_ENREF_42)) |
|  |  |  |  | *Cola gigantea* | Seeds | [Atolani et al. (2019](#_ENREF_27)), [Choi et al. (2007](#_ENREF_42)) |
| *β*-Caryophyllene (**40**) | Induces caspase-3, DNA fragmentation | -IC_50_ = 19, 63 and 27 µM against HCT116, HT-29 and PANC-1, respectively (in vitro) | 5-fluorouracil  -IC_50_ = 12.7 and 15 µM against HCT116, HT-29 respectively (in vitro)  Betulinic acid  -IC_50_ = 19, 4 µM against PANC-1 (in vitro) | *Brachychiton rupestris* | Leaves | [Thabet et al. (2020](#_ENREF_148)), [Fidyt et al. (2016](#_ENREF_51)), [Dahham et al. (2015](#_ENREF_8)) |

**NA**: not available

**TABLE S3 Bioactive metabolites in family Malvaceae and their anticancer mechanisms of action (Cont.)**

| **Compound name** | **Mechanism of action** | **Activity** | **Standard drug** | **Plant name** | **Part used** | **References** |
| --- | --- | --- | --- | --- | --- | --- |
| **Sterols & Terpenes (Cont.)** | | | | | | |
| Hibiscone C (**41**) | Reduces PI_3_K activity, Apoptosis | NA | NA | *Hibiscus tiliaceus* | Stems & twigs | [Μatsumoto et al. (2020](#_ENREF_165)), [Besley et al. (2017](#_ENREF_32)) |
| Loliolide (**42**) | Prevents cell damage induced by H_2_O_2_ | NA | NA | *Abelmoschus moschatus* | Aerial parts | [Sebastian et al. (2022](#_ENREF_126)), [Gangadhar et al. (2020](#_ENREF_52)) |
| Palmitic acid (**43**) | Affects DNA topoisomerase I | -Active at concentration 50 µg/ml against MOLT-4 (in vitro) | NA | *Brachychiton discolor* | Flowers | [Thabet et al. (2020](#_ENREF_148)), [Harada et al. (2002](#_ENREF_60)) |
| Phytol (**44**) | Increases cells in sub-G0 phase, decreases Bcl-2, increases Bax, activates caspase-3 & -9, antiangiogenic activity | -IC_50_ = 8.79, 77.85 and 60.7 µM against MCF-7, PC-3 and A549, respectively (in vitro) | NA | *Abelmoschus moschatus* | Aerial parts | [Sebastian et al. (2022](#_ENREF_126)), [Pejin et al. (2014](#_ENREF_106)), [Sakthivel et al. (2018](#_ENREF_121)) |
|  |  |  |  | *Corchorus olitorius* | Leaves | [Hassan et al. (2019](#_ENREF_62)), [Pejin et al. (2014](#_ENREF_106)), [Sakthivel et al. (2018](#_ENREF_121)) |
| *β*-Sitosterol (**45**) | Activates caspase enzymes, antiproliferative, apoptosis, scavenges ROS, decreases *β*-catenin & PCNA antigens, activates MAPK pathway, decreases bcl-2/bax ratio, DNA damage | - IC_50_ = 17.6 µg/ml against MCF-7 (in vitro)  -IC_50_ = 266.2 µM against COLO 320 DM (in vitro) | NA | *Abutilon hirtum* | Leaves | [HA (2001](#_ENREF_58)), [Singab et al. (2012](#_ENREF_134)), [Bin Sayeed and Ameen, (2015](#_ENREF_33)), [Baskar et al. (2010](#_ENREF_30)) |
|  |  |  |  | *Abutilon indicum* | Whole plant | [Gomaa et al. (2018](#_ENREF_56)), [Singab et al. (2012](#_ENREF_134)), [Bin Sayeed and Ameen, (2015](#_ENREF_33)), [Baskar et al. (2010](#_ENREF_30)) |

**NA**: not available

**TABLE S3 Bioactive metabolites in family Malvaceae and their anticancer mechanisms of action (Cont.)**

| **Compound name** | **Mechanism of action** | **Activity** | **Standard drug** | **Plant name** | **Part used** | **References** |
| --- | --- | --- | --- | --- | --- | --- |
| **Sterols & Terpenes (Cont.)** | | | | | | |
| *β*-Sitosterol (**45**) (Cont.) | Activates caspase enzymes, antiproliferative, apoptosis, scavenges ROS, decreases *β*-catenin & PCNA antigens, activates MAPK pathway, decreases bcl-2/bax ratio, DNA damage | - IC_50_ = 17.6 µg/ml against MCF-7 (in vitro)  -IC_50_ = 266.2 µM against COLO 320 DM (in vitro) | NA | *Althaea officinalis* | NA | [Kadhum](#_ENREF_73) et al. (2021), [Singab et al. (2012](#_ENREF_134)), [Bin Sayeed and Ameen, (2015](#_ENREF_33)), [Baskar et al. (2010](#_ENREF_30)) |
|  |  |  |  | *Cola gigantea* | Seeds | [Atolani et al. (2019](#_ENREF_27)), [Singab et al. (2012](#_ENREF_134)), [Bin Sayeed and Ameen, (2015](#_ENREF_33)), [Baskar et al. (2010](#_ENREF_30)) |
|  |  |  |  | *Corchorus olitorius* | Stems | [Ragasa et al. (2016](#_ENREF_111)), [Singab et al. (2012](#_ENREF_134)), [Bin Sayeed and Ameen, (2015](#_ENREF_33)), [Baskar et al. (2010](#_ENREF_30)) |
| *γ-*Sitosterol (**46**) | Stimulates apoptotic pathway, decreases *c-myc* oncogene expression | -IC_50_ = 8.3, 21.8 and 28.8 µg/ml against Caco-2, HepG2 and MCF-7, respectively (in vitro) | NA | *Abelmoschus moschatus* | Aerial parts | [Sebastian et al. (2022](#_ENREF_126)), [Endrini et al. (2014](#_ENREF_9)) |
| Stigmasterol (**47**) | Cell cycle arrest at G2/M phases, increases Bax, caspase-3 & -9, decreases Bcl-2, apoptosis, antiangiogenic | -IC_50_ = 15, 27.38 and 25.8 µM against SNU-1, MCF-7 and HepG2, respectively (in vitro) | NA | *Abutilon hirtum* | Leaves | [HA (2001](#_ENREF_58)), [Li et al. (2018](#_ENREF_82)) |
|  |  |  |  | *Althaea officinalis* | NA | [Kadhum](#_ENREF_73) et al. (2021), [Li et al. (2018](#_ENREF_82)) |
|  |  |  |  | *Corchorus olitorius* | Stems | [Ragasa et al. (2016](#_ENREF_111)), [Li et al. (2018](#_ENREF_82)) |
|  |  |  |  | *Cola gigantea* | Seeds | [Atolani et al. (2019](#_ENREF_27)), [Li et al. (2018](#_ENREF_82)) |

**NA**: not available

**TABLE S3 Bioactive metabolites in family Malvaceae and their anticancer mechanisms of action (Cont.)**

| **Compound name** | **Mechanism of action** | **Activity** | **Standard drug** | **Plant name** | **Part used** | **References** |
| --- | --- | --- | --- | --- | --- | --- |
| **Sterols & Terpenes (Cont.)** | | | | | | |
| Tanshindiol (**48**) | Reduces EZH2 activity | -GI_50_ = 4, 4.2 and 5.7 µM against PC-3, A549 and U87MG, respectively (in vitro) | GSK-126  -GI_50_ = 9.4, 18.7 and 28.5 µM against PC-3, A549 and U87MG, respectively (in vitro) | *Althaea rosea* | Flowers | [Abd El-Salam et al. (2016](#_ENREF_3)), [Woo et al. (2014](#_ENREF_155)) |
| Ursolic acid (**49**) | Increases ROS production, down-regulates Bcl-2 protein, apoptosis | -IC_50_ = 35.27 and 35 µM against MCF-7 and PC-3, respectively (in vitro) | NA | *Corchorus olitorius* | Roots | [Biswas et al., (2022](#_ENREF_5)),  [Mishra et al. (2016](#_ENREF_89)), [Kassi et al. (2007](#_ENREF_74)) |
| **Polysaccharides** | | | | | | |
| *β*-Glucan (**50**) | Antioxidant, chemopreventive | NA | NA | *Althaea officinalis* | Roots | [Deters et al. (2010](#_ENREF_46)), [Oliveira et al. (2013](#_ENREF_102)) |
| Pectin (**51**) | Apoptosis, DNA damage, antioxidant, anti-inflammatory | -IC_50_ of pectin gold nanoparticles = 8 and 2 µg/ml against MCF-7 and MDA-MB-231, respectively (in vitro) | NA | *Althaea officinalis* | Gums | Arfa (2022), [Deters et al. (2010](#_ENREF_46)), [Suganya et al. (2016](#_ENREF_142)), [Yan et al., (2023](#_ENREF_159)), [Teng et al. (2022](#_ENREF_147)) |

**NA**: not available

**TABLE S4 Bioavailability Radar mapping of the bioactive cytotoxic metabolites drug-likeness properties using SWISS ADME**

| 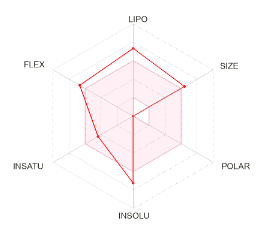  Bioavailability score = 0.85 | 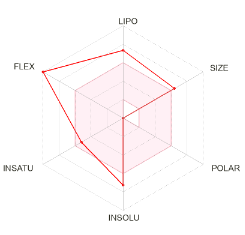  Bioavailability score = 0.85 | 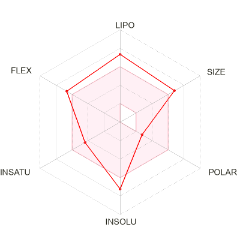  Bioavailability score = 0.85 | 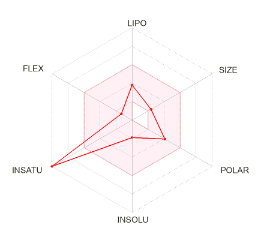  Bioavailability score = 0.56 |
| --- | --- | --- | --- |
| ***β*-Carotene** (**1**) | **Lycopene** (**2**) | **Zeaxanthin** (**3**) | **Caffeic acid** (**4**) |
| 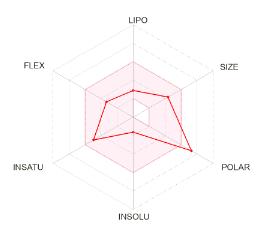  Bioavailability score = 0.11 | 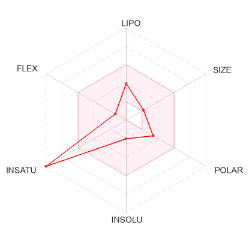  Bioavailability score = 0.85 | 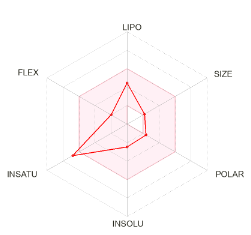  Bioavailability score = 0.55 | 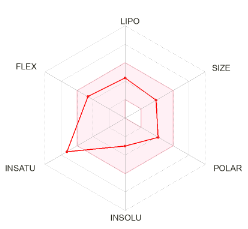  Bioavailability score = 0.55 |
| **Chlorogenic acid** (**5**) | ***p*-Coumaric acid** (**6**) | **Eugenol** (**7**) | ***N*-*trans*-Feruloyltyramine** (**8**) |

The favourable range for each drug-likeness feature is present in the pink area. (LIPO): lipophilicity (XLOGP3 (-0.7 to +5.0)); (INSOLU): insolubility in water (log S scale (not more than 6)); (FLEX): flexibility (rotatable bonds (not more than 9)); (SIZE): size (molecular weight (150 to 500 g/mol); (POLAR): (TPSA (20 to 130 A°^2^); (INSATU): in saturation (fraction of C hybridized in sp^3^ (0.25 to 1))

**TABLE S4Bioavailability Radar mapping of the bioactive cytotoxic metabolites drug-likeness properties using SWISS ADME (Cont.)**

| 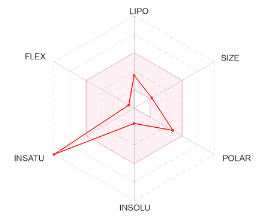  Bioavailability score = 0.56 | 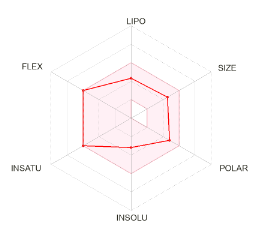  Bioavailability score = 0.55 | 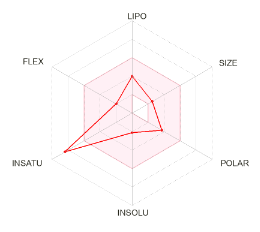  Bioavailability score = 0.55 | 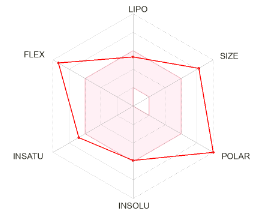  Bioavailability score = 0.17 |
| --- | --- | --- | --- |
| **Gallic acid** (**9**) | **Hibiscusamide** (**10**) | **Methyl caffeate** (**11**) | **Methyl-1,4,5-tri-*O*-caffeoyl quinate** (**12**) |
| 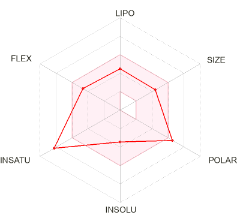  Bioavailability score = 0.56 | 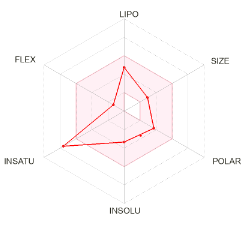  Bioavailability score = 0.55 | 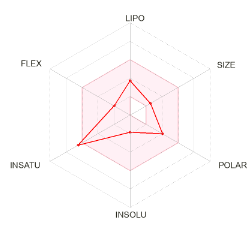  Bioavailability score = 0.56 | 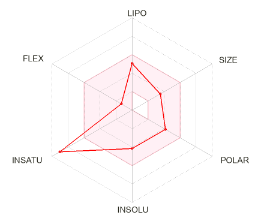  Bioavailability score = 0.55 |
| **Rosmarinic acid** (**13**) | **Syriacusin A** (**14**) | **Syringic acid** (**15**) | **Acacetin** (**16**) |

The favourable range for each drug-likeness feature is present in the pink area. (LIPO): lipophilicity (XLOGP3 (-0.7 to +5.0)); (INSOLU): insolubility in water (log S scale (not more than 6)); (FLEX): flexibility (rotatable bonds (not more than 9)); (SIZE): size (molecular weight (150 to 500 g/mol); (POLAR): (TPSA (20 to 130 A°^2^); (INSATU): in saturation (fraction of C hybridized in sp^3^ (0.25 to 1))

**TABLE S4 Bioavailability Radar mapping of the bioactive cytotoxic metabolites drug-likeness properties using SWISS ADME (Cont.)**

| 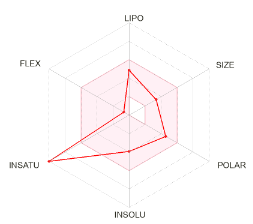  Bioavailability score = 0.55 | 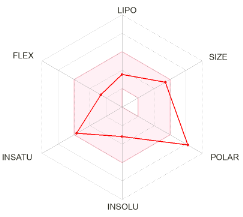  Bioavailability score = 0.17 | 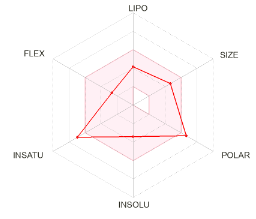  Bioavailability score = 0.56 | 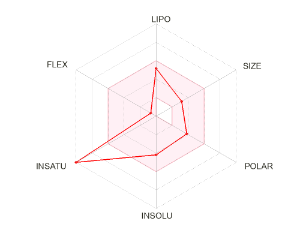  Bioavailability score = 0.55 |
| --- | --- | --- | --- |
| **Apigenin** (**17**) | **Astragalin** (**18**) | **Caicoine** (**19**) | **Chrysin** (**20**) |
| 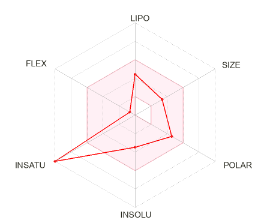  Bioavailability score = 0.55 | 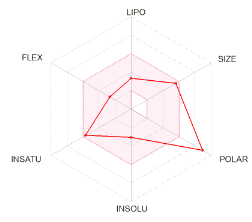  Bioavailability score = 0.17 | 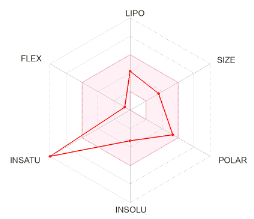  Bioavailability score = 0.55 | 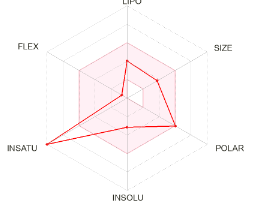  Bioavailability score = 0.55 |
| **Galangin** (**21**) | **Isoquercetin** (**22**) | **Kaempferol** (**23**) | **Quercetin** (**24**) |

The favourable range for each drug-likeness feature is present in the pink area. (LIPO): lipophilicity (XLOGP3 (-0.7 to +5.0)); (INSOLU): insolubility in water (log S scale (not more than 6)); (FLEX): flexibility (rotatable bonds (not more than 9)); (SIZE): size (molecular weight (150 to 500 g/mol); (POLAR): (TPSA (20 to 130 A°^2^); (INSATU): in saturation (fraction of C hybridized in sp^3^ (0.25 to 1))

**TABLE S4 Bioavailability Radar mapping of the bioactive cytotoxic metabolites drug-likeness properties using SWISS ADME (Cont.)**

| 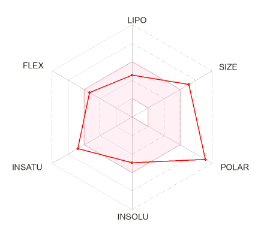  Bioavailability score = 0.17 | 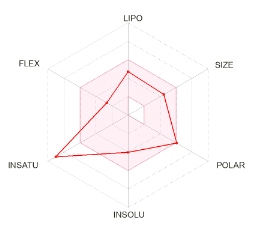  Bioavailability score = 0.56 | 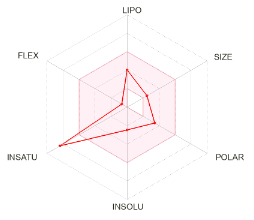  Bioavailability score = 0.55 | 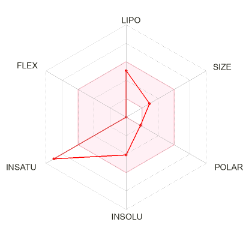  Bioavailability score = 0.55 |
| --- | --- | --- | --- |
| **Tiliroside** (**25**) | **Wissadulin** (**26**) | **Scopoletin** (**27**) | **Cryptolepine** (**28**) |
| 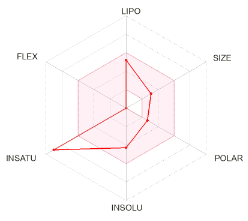  Bioavailability score = 0.55 | 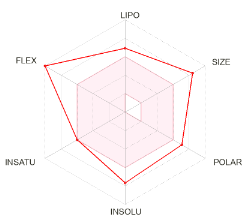  Bioavailability score = 0.17 | 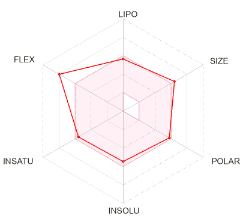  Bioavailability score = 0.55 | 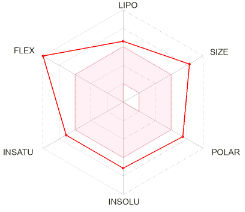  Bioavailability score = 0.17 |
| **Cryptolepinone** (**29**) | **Boehmenan** (**30**) | **Boehmenan H** (**31**) | ***threo*-Carolignan K** (**32**) |

The favourable range for each drug-likeness feature is present in the pink area. (LIPO): lipophilicity (XLOGP3 (-0.7 to +5.0)); (INSOLU): insolubility in water (log S scale (not more than 6)); (FLEX): flexibility (rotatable bonds (not more than 9)); (SIZE): size (molecular weight (150 to 500 g/mol); (POLAR): (TPSA (20 to 130 A°^2^); (INSATU): in saturation (fraction of C hybridized in sp^3^ (0.25 to 1))

**TABLE S4 Bioavailability Radar mapping of the bioactive cytotoxic metabolites drug-likeness properties using SWISS ADME (Cont.)**

| 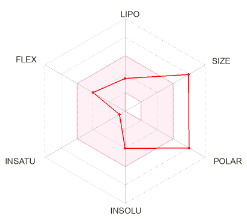  Bioavailability score = 0.17 | 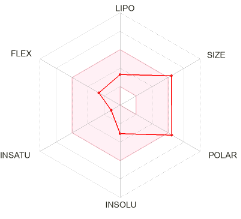  Bioavailability score = 0.55 | 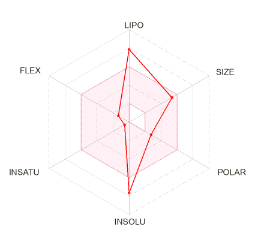  Bioavailability score = 0.55 | 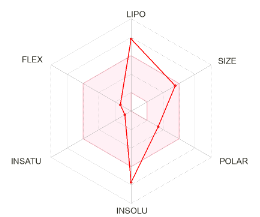  Bioavailability score = 0.85 |
| --- | --- | --- | --- |
| **Glucoevatromonosde** (**33**) | **Helveticoside** (**34**) | **Betulin** (**35**) | **Betulinic acid** (**36**) |
| 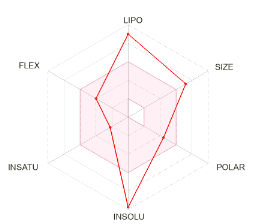  Bioavailability score = 0.17 | 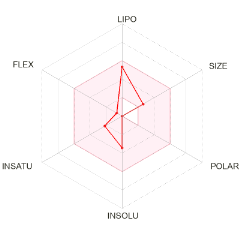  Bioavailability score = 0.55 | 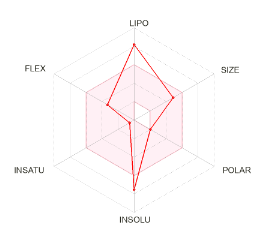  Bioavailability score = 0.55 | 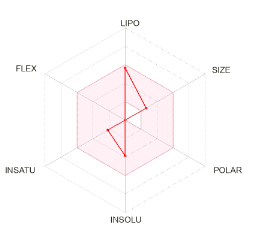  Bioavailability score = 0.55 |
| **Betulin-3-caffeate** (**37**) | ***δ*−Cadinene** (**38**) | **Campesterol** (**39**) | ***β*-Caryophyllene** (**40**) |

The favourable range for each drug-likeness feature is present in the pink area. (LIPO): lipophilicity (XLOGP3 (-0.7 to +5.0)); (INSOLU): insolubility in water (log S scale (not more than 6)); (FLEX): flexibility (rotatable bonds (not more than 9)); (SIZE): size (molecular weight (150 to 500 g/mol); (POLAR): (TPSA (20 to 130 A°^2^); (INSATU): in saturation (fraction of C hybridized in sp^3^ (0.25 to 1))

**TABLE S4 Bioavailability Radar mapping of the bioactive cytotoxic metabolites drug-likeness properties using SWISS ADME (Cont.)**

| 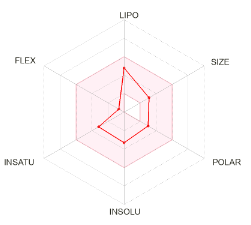  Bioavailability score = 0.55 | 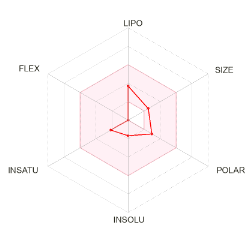  Bioavailability score = 0.55 | 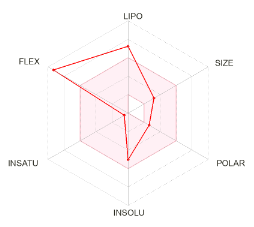  Bioavailability score = 0.85 | 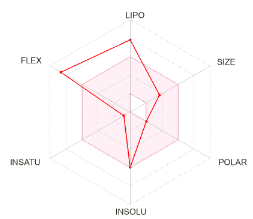  Bioavailability score = 0.55 |
| --- | --- | --- | --- |
| **Hibiscone C** (**41**) | **Loliolide** (**42**) | **Palmitic acid** (**43**) | **Phytol** (**44**) |
| 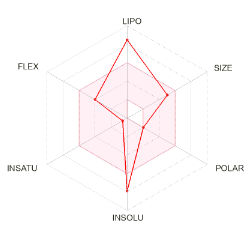  Bioavailability score = 0.55 | 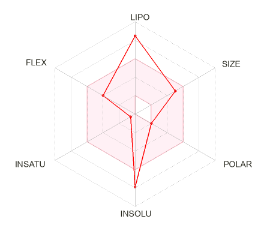  Bioavailability score = 0.55 | 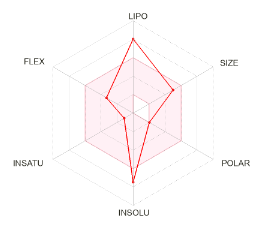  Bioavailability score = 0.55 | 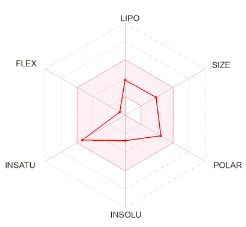  Bioavailability score = 0.55 |
| ***β*-Sitosterol** (**45**) | ***γ-*Sitosterol** (**46**) | **Stigmasterol** (**47**) | **Tanshindiol** (**48**) |

The favourable range for each drug-likeness feature is present in the pink area. (LIPO): lipophilicity (XLOGP3 (-0.7 to +5.0)); (INSOLU): insolubility in water (log S scale (not more than 6)); (FLEX): flexibility (rotatable bonds (not more than 9)); (SIZE): size (molecular weight (150 to 500 g/mol); (POLAR): (TPSA (20 to 130 A°^2^); (INSATU): in saturation (fraction of C hybridized in sp^3^ (0.25 to 1))

**TABLE S4 Bioavailability Radar mapping of the bioactive cytotoxic metabolites drug-likeness properties using SWISS ADME (Cont.)**

| 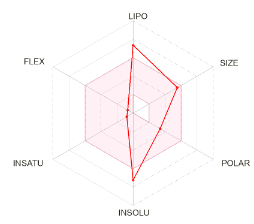  Bioavailability score = 0.85 | 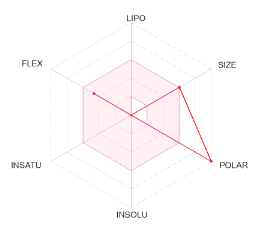  Bioavailability score = 0.17 | 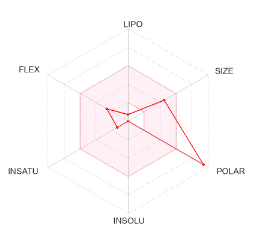  Bioavailability score = 0.11 | 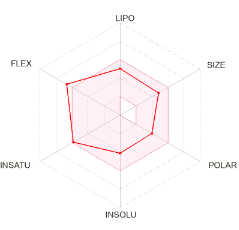  Bioavailability score = 0.55 |
| --- | --- | --- | --- |
| **Ursolic acid** (**49**) | ***β*-Glucan** (**50**) | **Pectin** (**51**) | **Erlotinib** |

The favourable range for each drug-likeness feature is present in the pink area. (LIPO): lipophilicity (XLOGP3 (-0.7 to +5.0)); (INSOLU): insolubility in water (log S scale (not more than 6)); (FLEX): flexibility (rotatable bonds (not more than 9)); (SIZE): size (molecular weight (150 to 500 g/mol); (POLAR): (TPSA (20 to 130 A°^2^); (INSATU): in saturation (fraction of C hybridized in sp^3^ (0.25 to 1))

**TABLE S5 Binding interactions of the most bioactive compounds against EGFR kinase (8A27)**

| **Compound** | **Hydrophobic** | **Hydrogen bond** | **Salt bridge** |
| --- | --- | --- | --- |
| **3** | LEU 718 | - | - |
|  | Val 726 |  |  |
|  | Val 726 |  |  |
|  | ALA 743 |  |  |
|  | LEU 799 |  |  |
|  | ARG 841 |  |  |
|  | LEU 844 |  |  |
|  | LEU 844 |  |  |
|  | LYS 879 |  |  |
|  | TRP 880 |  |  |
|  | LYS 913 |  |  |
| **12** | LEU 718 | LEU 718 | - |
|  | LEU 718 | LYS 728 |  |
|  | VAL 726 | LYS 745 |  |
|  | VAL 726 | ARG 803 |  |
|  | LYS 728 |  |  |
|  | ALA 743 |  |  |
|  | LYS 745 |  |  |
|  | THR 790 |  |  |
|  | LEU 792 |  |  |
|  | LEU 799 |  |  |
|  | ARG 841 |  |  |
|  | LEU 844 |  |  |
|  | LEU 844 |  |  |
| **16** | VAL 726 | LYS 745 | - |
|  | LEU 777 | MET 766 |  |
|  | LEU 844 | THR 854 |  |
| **18** | - | ALA 722 | - |
|  |  | PHE 723 |  |
|  |  | THR 790 |  |
| **20** | VAL 726 | LYS 745 | - |
|  | VAL 726 | THR 854 |  |
|  | THR 790 |  |  |
|  | LEU 844 |  |  |
| **22** | ALA 743 | SER 720 | - |
|  | LEU 844 | THR 854 |  |
|  | THR 854 |  |  |
| **25** | LEU 718 | PHE 723 | - |
|  | PHE 723 | GLY 724 |  |
|  | VAL 726 | LYS 745 |  |
|  | ASP 855 | THR 790 |  |
|  | LEU 858 | MET 793 |  |
|  |  | ASP 800 |  |
| **29** | LEU 777 | LYS 745 | - |
|  | LEU 777 | ASP 855 |  |
|  | THR 790 | ASP 855 |  |
|  | LEU 858 |  |  |
|  | LEU 858 |  |  |
| **30** | ALA 722 | LYS 745 | - |
|  | PHE 723 |  |  |
|  | PHE 723 |  |  |
|  | VAL 726 |  |  |
|  | ALA 743 |  |  |
|  | LYS 745 |  |  |
|  | LYS 745 |  |  |
|  | LYS 745 |  |  |
|  | LEU 777 |  |  |
|  | LEU 788 |  |  |
|  | LEU 788 |  |  |
|  | THR 790 |  |  |
|  | LEU 844 |  |  |
|  | LEU 858 |  |  |
|  | LEU 858 |  |  |
|  | LEU 858 |  |  |
| **31** | LYS 745 | SER 720 | LYS 745 |
|  | MET 766 | LYS 745 |  |
|  | LEU 777 | ASP 855 |  |
|  | LEU 788 | PHE 856 |  |
|  | PHE 856 | GLY 857 |  |
|  | LEU 858 |  |  |

**TABLE S5 Binding interactions of the most bioactive compounds against EGFR kinase (8A27)**

| **Compound** | **Hydrophobic** | **Hydrogen bond** | **Salt bridge** |
| --- | --- | --- | --- |
| **33** | LEU 718 | MET 793 | - |
|  | VAL 726 | ARG 841 |  |
|  | VAL 726 | ARG 841 |  |
|  | ALA743 | VAL 876 |  |
|  | ARG 841 | VAL 876 |  |
| **39** | LEU 747 | GLU 758 | **-** |
|  | ILE 759 |  |  |
|  | GLU 762 |  |  |
|  | ALA 763 |  |  |
|  | LEU 777 |  |  |
|  | LEU 777 |  |  |
|  | THR 790 |  |  |
| **45** | PHE 723 | GLY 857 | **-** |
|  | PHE 723 |  |  |
|  | THR 751 |  |  |
|  | GLU 758 |  |  |
|  | ILE 759 |  |  |
|  | ILE 759 |  |  |
|  | LEU 788 |  |  |
|  | LEU 858 |  |  |
|  | LEU 858 |  |  |
| **47** | LYS 745 | **-** | **-** |
|  | LYS 745 |  |  |
|  | LEU 777 |  |  |
|  | LEU 788 |  |  |
|  | THR 790 |  |  |
|  | LEU 844 |  |  |
|  | THR 845 |  |  |
| **Erlotinib** | VAL 726 | LYS 745 | **-** |
|  | ALA 743 |  |  |
|  | LYS 745 |  |  |
|  | LYS 745 |  |  |
|  | LEU 788 |  |  |
|  | THR 790 |  |  |
|  | ARG 841 |  |  |
|  | LEU 844 |  |  |
